# Quantifying Global Wetland Methane Emissions With In Situ Methane Flux Data and Machine Learning Approaches

**DOI:** 10.1029/2023EF004330

**Published:** 2024-10-31

**Authors:** Shuo Chen, Licheng Liu, Yuchi Ma, Qianlai Zhuang, Narasinha J. Shurpali

**Affiliations:** ^1^ Department of Earth, Atmospheric, Planetary Sciences Purdue University West Lafayette IN USA; ^2^ Department of Earth System Science and Center on Food Security and the Environment Stanford University Stanford CA USA; ^3^ Department of Agronomy Purdue University West Lafayette IN USA; ^4^ Production Systems Unit Natural Resources Institute Finland (Luke) Maaninka Finland

## Abstract

Wetland methane (CH_4_) emissions have a significant impact on the global climate system. However, the current estimation of wetland CH_4_ emissions at the global scale still has large uncertainties. Here we developed six distinct bottom‐up machine learning (ML) models using in situ CH_4_ fluxes from both chamber measurements and the Fluxnet‐CH_4_ network. To reduce uncertainties, we adopted a multi‐model ensemble (MME) approach to estimate CH_4_ emissions. Precipitation, air temperature, soil properties, wetland types, and climate types are considered in developing the models. The MME is then extrapolated to the global scale to estimate CH_4_ emissions from 1979 to 2099. We found that the annual wetland CH_4_ emissions are 146.6 ± 12.2 Tg CH_4_ yr^−1^ (1 Tg = 10^12^ g) from 1979 to 2022. Future emissions will reach 165.8 ± 11.6, 185.6 ± 15.0, and 193.6 ± 17.2 Tg CH_4_ yr^−1^ in the last two decades of the 21st century under SSP126, SSP370, and SSP585 scenarios, respectively. Northern Europe and near‐equatorial areas are the current emission hotspots. To further constrain the quantification uncertainty, research priorities should be directed to comprehensive CH_4_ measurements and better characterization of spatial dynamics of wetland areas. Our data‐driven ML‐based global wetland CH_4_ emission products for both the contemporary and the 21st century shall facilitate future global CH_4_ cycle studies.

## Key Points

- A multi‐model ensemble is conducted to quantify the global wetland methane emissions
- Estimated global wetland methane emissions are 146.6 ± 12.2 Tg CH_4_ yr^−1^ during 1979–2022 and will increase by 13.1% (SSP126), 26.6% (SSP370), and 32.0% (SSP585) by 2080–2099
- Temperature, elevation, soil organic matter, and surface downward solar radiation are the most important features in six machine learning wetland methane emission models

## 1 Introduction

Methane (CH_4_), the second most important greenhouse gas following carbon dioxide (CO_2_), has been responsible for 20% of the observed warming since pre‐industrial times (Ciais et al., 2013). According to the IPCC Sixth Assessment Report, the global warming potential of CH_4_ from non‐fossil fuel sources is 27.2 times that of CO_2_ in the 100‐year time period (Calvin et al., 2023). Notably, the atmospheric CH_4_ concentration has increased from 700 ppb in the pre‐industrial period to 1,920 ppb in 2023 (Lan et al., 2022). The dynamics of atmospheric CH_4_ concentration have a high variability. It increased rapidly in the early 1980s, slowed down between 1999 and 2006, and has been rising again since 2007 and became one of the highest ever observed in 2021 (Dlugokencky et al., 2009; Nisbet et al., 2014; Saunois et al., 2016, pp. 2000–2012; Schaefer et al., 2016; Z. Zhang et al., 2018). This variability in atmospheric CH_4_ concentration is influenced by the climatic sensitivity of wetlands which contribute 30%–40% of total CH_4_ emissions and this significant role is expected to continue into the future (Koffi et al., 2020; Z. Zhang et al., 2017). Thus, it is vital to understand the dynamics of wetland CH_4_ emissions.

Currently, there have been three popular approaches employed to estimate CH_4_ emissions from wetlands across various scales: (a) bottom‐up approaches, which use in situ CH_4_ measurement and process‐based models to quantify CH_4_ emissions, (b) top‐down approaches, which estimate CH_4_ emissions by measuring atmospheric concentrations and then using inverse modeling to trace these concentrations back to their sources and (c) data‐driven approaches, which leverage extensive data sets to empirical models and predict methane emissions based on observed patterns and correlations (Anderson et al., 2010; Arneth et al., 2010; Kirschke et al., 2013; Saunois et al., 2020; Q. Zhu et al., 2014). Bottom‐up and top‐down approaches have been developed previously to quantify wetland CH_4_ emissions (Cao et al., 1996; Li, 2000; Meng et al., 2012; Saunois et al., 2020, pp. 2000–2017; Y. Zhang et al., 2002; Z. Zhang et al., 2017; Zhuang et al., 2004; Q. Zhu et al., 2014). However, bottom‐up approaches are usually constrained by the complex parameter optimization process, the need for detailed local data, and the challenge of accurately extrapolating these data to larger scales. On the other hand, top‐down approaches usually struggle with accurately pinpointing specific emission sources due to the diffuse nature of atmospheric methane.

In contrast, data‐driven approaches can efficiently handle large volumes of data from various sources, including flux measurements, climate data, and remote sensing observations, and capture the complex nonlinear relationship between flux measurements and environmental variables (Bomers et al., 2019; Delon et al., 2007; Dupont et al., 2008; S. Liu et al., 2016, 2016; X. Zhu et al., 2013). In recent years, the use of ML models to predict CH_4_ emission has become increasingly popular (Irvin et al., 2021; Kim et al., 2020; Luo et al., 2023; Yuan et al., 2022). For example, Yuan et al. developed a causality‐enabled machine‐learning model for CH_4_ emission across global wetlands (Yuan et al., 2022). A recent study upscaled eddy covariance methane fluxes to a global scale using data from 43 FLUXNET‐CH_4_ measurement sites and a random forest model (McNicol et al., 2023). However, it only adopted eddy covariance (EC) flux data without using the chamber measurement data. At present, besides the accumulated eddy covariance flux measurements through FLUXNET, many chamber‐based measurements have also been accumulated in the published literature. The combination of chamber and tower flux data provides opportunities for ML models to better estimate global wetland methane emissions (Bansal et al., 2023; Kuhn et al., 2021; McNicol et al., 2023; Turetsky et al., 2014). Making use of all available data measured using both methods increases the input data space to better capture the inherent patch‐scale effects (K. Xu et al., 2017) and extends the temporal representativeness of flux data (Chu et al., 2017). Moreover, there are only a few future projection studies of wetland methane emissions (Bansal et al., 2023; Z. Zhang et al., 2017).

In addition, current quantifications on global natural wetland CH_4_ emissions still have large uncertainties. The global estimates of CH_4_ emissions from natural wetlands during 2000–2017 range from 100 to 217 Tg CH_4_ yr^−1^ when using different approaches and have a larger uncertainty in comparison with anthropogenic emissions (L. Liu et al., 2020; Saunois et al., 2020). The Wetland CH_4_ Inter‐Comparison of Models Project (WETCHIMP) (Melton et al., 2013) and Atmospheric Chemistry and Transport modeling (WetCHARTS) (Bloom et al., 2017) projects have provided insights into uncertainty in bottom‐up and top‐down models, respectively. The uncertainty in these estimates results from many sources including model structures, assumptions, and parameterization (McNicol et al., 2023; Saunois et al., 2020). As an emerging method, there are currently few studies on evaluating and reducing the uncertainty of estimates from different machine learning models. Fortunately, the multi‐model ensemble (MME) approach is a promising approach to reducing model uncertainty and has been considered as an effective method to minimize uncertainty in climate change simulation in climate change research in other fields (Rosenzweig et al., 2014).

In this study, we have developed an MME approach integrating six advanced ML models, chamber and EC flux tower data, and climate forcing to estimate global wetland methane emissions in contemporary and future scenarios. Specifically: (a) We organized in situ CH_4_ flux of 35 chamber sites and 47 eddy covariance sites of various wetland ecosystems around the globe from 1988 to 2019; (b) We have developed an MME approach integrating six advanced ML models, including decision tree (DT), random forest (RF), extreme gradient boosting (XGB), artificial neural network (ANN), Gated Recurrent Units (GRU) and Long Short‐Term Memory (LSTM), to capture nonlinear relation between CH_4_ fluxes and key environmental variables; (c) The developed MME was extrapolated to estimate global wetland CH_4_ emissions during the historical and future periods, driven by the spatially explicit data of climate, hydrology, and soil properties; (d) We have also conducted rigorous analysis on methane emission responses to environmental variables to help understand the mechanisms controlling the spatial and temporal patterns of CH_4_ emissions. This study represents the first intercomparison of advanced ML models applied to wetland emission modeling and will shed light on the next‐generation global methane budget estimations leveraging recent ML techniques properly.

## 2 Materials and Methods

### 2.1 Overview

**Figure 1:**
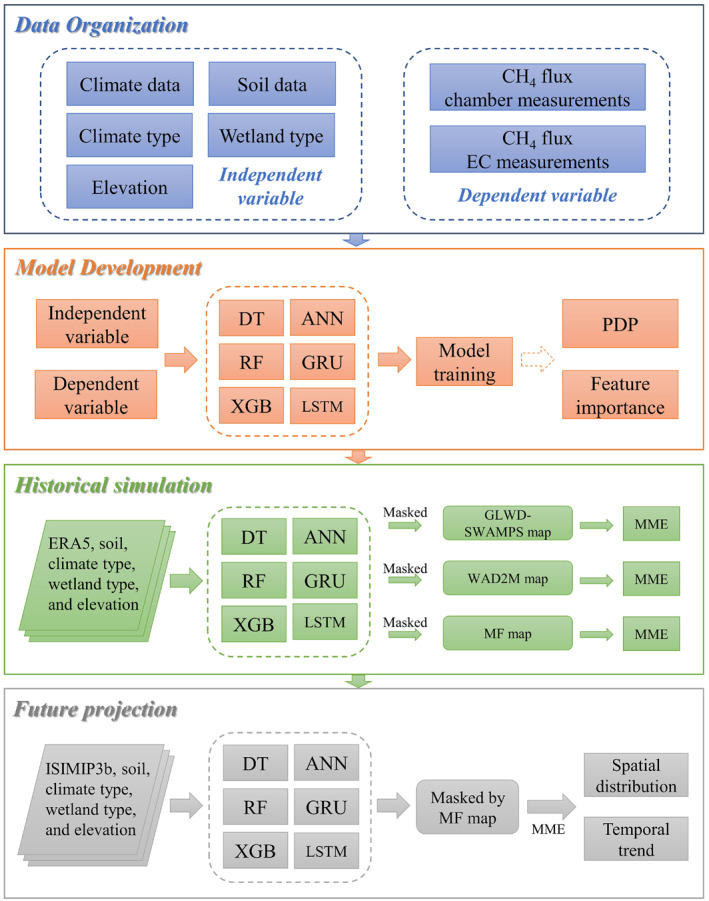
Schematic diagram of upscaling wetland methane emission from site level to global scale. Machine learning methods include decision tree (DT); random forest (RF); extreme gradient boosting (XGB); artificial neural network (ANN); Gated Recurrent Units (GRU); and Long Short‐Term Memory (LSTM). PDP refers to partial dependency plot. MF map refers to static inundation area fraction from Matthews and Fung (1987). WAD2M map refers to WAD2M transient wetland inundation area fraction data. GLWD‐SWAMPS map refers to GLWD‐SWAMPS transient wetland inundation area fraction data. MME refers to multiple model ensembles. ERA5 refers to historical climate data from the European Centre for Medium‐Range Weather Forecasts (ECMWF) Reanalysis v5 (Hersbach et al., 2020). ISIMIP3b refers to the future climate data of 5 CMIP6 GCMs bias‐corrected and spatial‐downscaled by Inter‐Sectoral Impact Model Intercomparison Project phase 3b (Lange, 2019).

We first organized in situ CH_4_ flux data from 35 chamber sites and 47 eddy covariance sites of various wetland ecosystems around the globe from 1988 to 2019. The data were used to train six ML models to estimate CH_4_ flux. We then developed the MME based on six ML models and extrapolated it to the global scale using gridded climate data, soil properties, elevation, wetland types, climate types, and wetland inundation area to estimate natural wetland methane emissions during 1979–2022 (Figure 1). We have also conducted the uncertainty analysis and analyzed how methane emission responds to environmental variables for this period. Finally, we used the multi‐model ensemble (MME) models to predict future wetland methane emissions during 2015–2100.

### 2.2 Data

**Figure 2:**
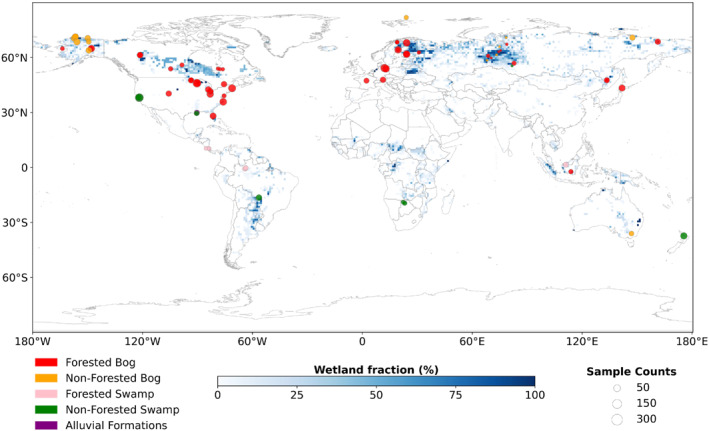
Location, wetland type, and size of 82 globally distributed wetland sites. Symbol sizes represent the number of CH_4_ emission samples in each site. Symbol colors represent the wetland type. The wetland fraction was derived from the MF static wetland distribution map.

Site‐level CH_4_ flux data were obtained from 35 chamber sites and 47 eddy covariance sites of various wetland ecosystems around the globe from 1988 to 2019 (Figure 2). Among them, 10 sites are from the tropics (23.4°S–23.4°N). The eddy covariance measurements are collected from FLUXNET‐CH_4_ (Delwiche et al., 2021) while chamber measurements are collected from peer‐reviewed literature (Table S1 in Supporting Information S1). The raw CH_4_ flux data were recorded at different time scales, varying from half‐hourly to monthly. They were unified into monthly values by averaging non‐monthly data within a month and aggregating them into monthly values.

Environmental variables include climate data, soil properties, elevation, wetland types, and climate types. Climate data, especially temperature and precipitation, have proven to be important predictors of CH_4_ emissions in previous studies (L. Liu et al., 2020; McNicol et al., 2023; Yuan et al., 2022). Soil properties and elevation were also selected due to their significant influence on wetland hydrology and soil microbial activity. For example, soil pH and the availability of nutrients like nitrogen and phosphorus can affect microbial communities and their metabolic pathways, including those involved in methanogenesis and CH_4_ oxidation.

Dynamic climate data includes precipitation (PREC), surface air temperature (TAIR), surface downward solar radiation (SOLAR), and relative humidity (RH). Site‐level climate data were first collected from their original site records if available. Otherwise, the missing climate data of each site were complemented using the ERA5 reanalysis climatic data set (Hersbach et al., 2020). Gridded historical climate data were obtained from the ERA5 monthly averaged data set (Hersbach et al., 2020). They were provided as monthly values at 0.25° × 0.25° spatial resolution from 1940 to the present. In this research, we extracted the data from 1979 to 2022 and resampled them to 0.5° × 0.5° spatial resolution. Gridded future projection of climate data was obtained from the Inter‐Sectoral Impact Model Inter‐comparison Project phase 3b (ISIMIP3b) (Lange, 2019). ISIMIP3b provided the bias‐corrected and spatial‐downscaled climatic data of GFDL‐ESM4, IPSL‐CM6A‐LR, MPI‐ESM1–2‐HR, MRI‐ESM2‐0, and UKESM1‐0‐LL, which participated in Coupled Model Inter‐comparison Project Phase 6 (CMIP6). ISIMIP3b climatic data were provided as daily values at 0.5° × 0.5° spatial resolution from 1850 to 2100 (Lange, 2019). In this research, we extracted the data from 1995 to 2100 and further resampled them to monthly values. The historical and future climate type data were obtained from Köppen‐Geiger climate classification maps (Beck et al., 2023).

Soil properties, elevation, and wetland type are static variables. They were assumed to be constant over time in model development, historical simulation, and future projection. Soil properties include the bulk density of the soil (BULK), soil C/N ratio (CNRT), soil organic carbon content (ORGC), soil pH (PHAQ), soil sand (SDTO), and silt content (STPC). Site‐level soil properties were first collected from their original site records if available. Otherwise, the missing soil properties of each site were complemented using the International Soil Reference and Information Centre World Inventory of Soil Emission Potentials spatial soil database (WISE30sec) (Batjes, 2016). WISE30sec provided gridded soil properties of 7 layers for depths 0–200 cm, layers D1–D5 each represent 20 cm (0–100 cm), and layers D6–D7 each represent 50 cm (100–200 cm). We calculated the average soil properties of all layers. Take BULK as an example,

$$\text{BULK}=\frac{\text{BULK}\_D1*20\text{cm}+\text{BULK}\_D2*20\text{cm}+\text{BULK}\_D3*20\text{cm}+...+\text{BULK}\_D6*50\text{cm}+\text{BULK}\_D7*50\text{cm}}{\left(20+20+20+20+20+50+50\right)\text{cm}}.$$

The site‐level elevation (clelev) was first collected from their original site records if available. Otherwise, missing clelev was complemented using the Global 1 arc second Digital Elevation Model (GDEM) (Toutin, 2002). The gridded soil properties from WISE30sec and clelev from GDEM were resampled to 0.5° × 0.5° spatial resolution and used as input in historical simulation and future projection. Site‐level wetland types were determined by the site description and the classification (Matthews & Fung, 1987). Wetland types were categorized to forested bog, forested swamps, non‐forested bogs, non‐forested swamps and alluvial formations (Figure 2). Gridded wetland types were generated similarly by vegetation distribution and wetland distribution (Matthews & Fung, 1987; Melillo et al., 1993).

### 2.3 Model Development

The in situ CH_4_ data was paired with site‐level climate data, soil properties, elevation, wetland types, and climate types used for ML model development. We applied decision tree (DT), random forest (RF), extreme gradient boosting (XGB), artificial neural network (ANN), Gated Recurrent Units (GRU), and Long Short‐Term Memory (LSTM) to establish the non‐linear relationship between environmental variables and CH_4_ fluxes.

DT, RF, and XGB are all tree‐based (TB) models. DT is a non‐parametric supervised learning method capable of deriving decision rules from a series of data characterized by features and labels (Breiman et al., 1984). These rules are presented in a tree‐like graphical structure, utilized to address classification and regression problems. RF and XGB are ensemble algorithms, which consider the modeling results of multiple evaluators (trees) and obtain a comprehensive result after aggregating the result from each tree. They thereby achieved better regression performance compared to a single tree. RF constructs multiple independent evaluators and then determines the result of the ensemble evaluator based on an average or majority voting principle (Breiman, 2001). XGBoost employs a boosting methodology, where it sequentially constructs decision trees, with each subsequent tree designed to rectify the errors of its predecessor (T. Chen & Guestrin, 2016).

ANN, GRU, and LSTM models are all neural network (NN) models. NN models imitate the neural networks present in the human brain. It usually consists of interconnected nodes (neurons), an input layer, hidden layers, and an output layer. The connections between neurons have their own weights, which are adjusted during the learning process. Both GRU and LSTM are recurrent neural networks. They are designed to learn long‐term dependencies in time‐series data and remember information for prolonged periods of time (Hochreiter & Schmidhuber, 1997). Recurrent neural networks may do better than other ML models for time series prediction because time‐series features have temporal dependencies, which signifies the influence that past observations have on future ones. Recurrent neural networks are able to retain the memory of past inputs, capturing temporal dependencies more effectively than other machine learning models. Generally, the GRU cell has 2 gates (reset and update gates) while the LSTM cell has three gates (forget, input, and output gates). These gates control the information flow and decide which information to proceed and which to forget.

We used the grid search method on a set of hyperparameters to determine the optimal ML model structures (Table S2 in Supporting Information S1). As a result, for DT, the maximum depth of the tree (max_depth) was set to 15, and the minimum number of samples required to split an internal node (min_samples_split) was set to 12. To prevent overfitting in the trained DT, the maximum of levels of nodes for a tree to grow is 15 and if the number of samples in the split leaf nodes is less than 12, the growth will also stop. For RF, the number of estimators (n_estimators) was set to 50, max_depth was set to 10 and min_samples_split was set to 6. Besides max_depth and min_samples_split, setting n_estimators to 50 also helps avoid overfitting because the ensemble model will only consist of 50 individual decision trees. For XGB, n_estimators were set to 50, max_depth was set to none, and the learning rate (learning_rate) was set to 0.05. The relatively smaller learning rate (0.05) can contribute to better generalization. For NN models, the best‐performing ANN model has 4 hidden layers and each has 256, 128, 64, and 32 neurons with 1,000 epochs. The GRU model has 3 hidden layers and each has 256, 128, and 64 neurons with 2000 epochs. The LSTM model has 2 hidden layers and each has 256 and 128 neurons with 2,000 epochs. The Rectified Linear Unit (ReLU) was used as the activation function for three NN models.

We used the 10‐fold cross‐validation method to validate the model performance. Specifically, the entire data set was partitioned into 10 equal subsets; throughout the validation process, the model was trained on 9 subsets (or folds) and validated on the remaining one. This process was iterated 10 times, with each fold serving once as the validation set. After the 10 iterations, the average validation accuracy was calculated. This method facilitates a comprehensive assessment of the model's performance, minimizing both bias and variance and ensuring a more generalized model that can provide reliable predictions on unseen data. The coefficient of determination (*R* ^2^), root‐mean‐square error (RMSE), and relative RMSE ($\text{rRMSE}=\sqrt{\frac{\sum_{i=1}^{N}{\left(y_{i}-{\hat{y}}_{i}\right)}^{2}}{\sum_{i=1}^{N}{\left(y_{i}\right)}^{2}}}$) between observed (*y*_*i*_) and predicted (${\hat{y}}_{i}$) methane flux were calculated to assess the predictive accuracy.

We used the leave‐one‐year‐out method to evaluate the interannual variability and seasonal cycles. Specifically, we trained the models on data from all years except one, which is held out as the test set, and repeated this process such that each year serves as the test set exactly once. For interannual variability, we compared the standard deviation (STD) of MME‐predicted and field‐measured monthly methane emissions within the testing year, using *R* ^2^, RMSE, and relative RMSE. For seasonal cycles, we plotted the time series of MME predicted and measured methane emissions for every testing year.

In addition, we used the permutation feature importance approach on 6 ML models to analyze the contribution of each environmental variable in predicting the CH_4_ emission (Breiman, 2001; Grömping, 2009). The partial dependence plots (PDP) were used to analyze the response of the methane emission to each predictor variable (S. Chen et al., 2022; P. Zhu et al., 2021).

After validation, we developed the multi‐model ensemble (MME) by averaging the estimates of six ML models trained by the full set of data to upscale CH_4_ emissions. MME can cancel out the noise and errors in individual models and deliver more robust estimates (Feng et al., 2020). Before conducting historical and future simulations, we evaluated the representativeness of training data by calculating the ratio of wetland grids in historical and future climate forcing that exceeded the range of climatic variables in the training data. The training data set can fully cover the variable space of TAIR, PREC, SOLAR, and RH within global wetlands in the historical period (1979–2022). For the future period (2015–2100), it can fully cover the variable space of PREC, SOLAR, and RH as well as more than 97% of TAIR in the 5 GCMs (Tables S3 and S4 in Supporting Information S1).

### 2.4 Historical Simulation

To study the spatial distribution and temporal dynamics of historical CH_4_ emission at the global scale, the gridded ERA5 climate data, WISE30sec soil properties, GDEM elevation data, wetland types, and climate types were used to drive the MME to conduct historical simulation on CH_4_ flux at 0.5° × 0.5° spatial resolution from 1979 to 2022. The results were masked by the static wetland distribution map (Matthews & Fung, 1987). The estimated CH_4_ flux was further aggregated to annual total emissions and summarized by latitude zones. To compare the differences in results driven by transient and static wetland inundation areas, we set up another experiment. With other settings unchanged, we used two transient wetland inundation area fraction data, named GLWD‐SWAMPS (Poulter et al., 2017) and WAD2M Version 2.0 (Z. Zhang et al., 2021). GLWD‐SWAMPS provided monthly wetland inundation areas from 2000 to 2012 and were sourced from Surface WAter Microwave Product Series (SWAMPS) (Schroeder et al., 2015) and Global Lakes and Wetlands Database (GLWD) (Lehner & Döll, 2004). The Wetland Area and Dynamics for Methane Modeling (WAD2M) Version 2.0 provided monthly wetland inundation areas from 2000 to 2020 and was developed based on SWAMPS (Z. Zhang et al., 2021). It was used in the Global Carbon Project (GCP) bottom‐up model ensemble (Saunois et al., 2020) and the only current ML‐based global wetland methane emission upscaling product (McNicol et al., 2023). MF static wetland map indicates a larger area of high wetland fraction in the West of the Ural Mountains than GLWD‐SWAMPS and WAD2M (Figure S1 in Supporting Information S1). GLWD‐SWAMPS presented a larger wetland fraction in the Saharan regions than MF and WAD2M. MF static wetland map is overall closer to WAD2M than GLWD‐SWAMPS.

To quantify the biases caused by the static wetland map assumptions, we calculated the difference in the annual wetland emissions between the one masked by the transient wetland inundation map (dynamic_*t*_) and the one masked by the static wetland inundation map (static_*t*_) during the overlapping years:

$$\text{Bias}=\frac{1}{\text{years}}\sum_{i=t}^{\text{years}}\left({\text{static}}_{t}\;-{\text{dynamic}}_{t}\right)$$

where years is the amount of overlapping years and *t* is the specific year.

### 2.5 Future Projection

To study the spatial distribution and temporal dynamics of future CH_4_ emission at the global scale, the gridded ISIMIP3b climate data, WISE30sec soil properties, GDEM elevation data, wetland types, and climate types were used to drive the MME to conduct projection on CH_4_ flux at 0.5° × 0.5° spatial resolution from 1995 to 2100, divided into baseline (1995–2014) and future period (2015–2100). Specifically, five GCMs from ISIMIP3b were used to drive the MME. We chose ISIMIP3b because it bias‐adjusted and statistically downscaled (BASD) historic and future climate data from the latest GCMs (CMIP6). Also, ISIMIP3b has been widely used in other related studies (D. Xu et al., 2024), which will further enable intercomparison. Before BASD, ISIMIP3b evaluated the performance of more than 20 candidate CMIP6 GCMs and found climate variables including air temperature, precipitation, relative humidity, and short‐wave radiant in GFDL‐ESM4, MPI‐ESM1‐2‐HR, MRI‐ESM2‐0, IPSL‐CM6A‐LR, and UKESM1‐0‐LL outperform the other GCMs in the historical period. After BASD, these five selected GCMs matched historical observation better and preserved the original warming signal in CMIP6 (Lange, 2021). They can well represent the whole range of GCM ensemble simulation as GFDL‐ESM4, MPI‐ESM1‐2‐HR, and MRI‐ESM2‐0 are low climate sensitivity while IPSL‐CM6A‐LR and UKESM1‐0‐LL are high climate sensitivity (Lange, 2019). Then, we conducted future projections on future periods under SSP126, SSP370, and SSP585 scenarios. SSP126 depicts a sustainable world with low emissions and a 1.8°C temperature rise by 2100. SSP370 depicts higher emissions with regional conflicts, leading to a 2.6–4.7°C temperature increase. SSP585 depicts a fossil‐fuel‐reliant world, resulting in a significant temperature rise of 3.3–5.7°C by 2100 (O’Neill et al., 2016).

To evaluate the spatial and temporal change of future CH_4_ emissions, we calculated the spatial difference between the 20‐year average of the last 2 decades of this century (2080–2099) and the baseline (1995–2014). Then we summarized the annual CH_4_ emissions from 1995 to 2100 under SSP126, SSP370, and SSP585 scenarios and presented spatial maps of future trends of CH_4_ and the associated uncertainties by different MLs and different GCMs. Specifically, we quantitatively assessed the temporal trend of annual methane emissions for each grid cell by applying linear regression analysis: *y* = *β*_0_ + *β*_1_ × Year_*t*_ + *ε*_*t*_. Here, *y* is the annual methane emissions in year *t*, *β*_0_ is the intercept of the linear model, *β*_1_ is the slope, representing the trend in methane emissions over time, Year_*t*_ is the year at time *t*, and *t*, and *ε*_*t*_ is the error term. The ML model uncertainty was calculated from the STD of methane emissions generated from different ML models while GCMs uncertainty was calculated from the STD of methane emissions driven by different GCM climate forcing.

## 3 Results

### 3.1 Predictive Modeling

#### 3.1.1 Model Performance

**Figure 3:**
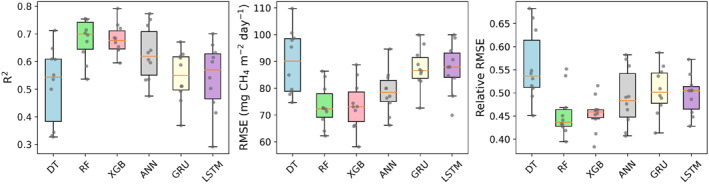
The comparisons of overall prediction accuracy from 10‐fold cross‐validation for methane emissions. The upper and bottom lines of the box represent the 25th and 75th percentile of the *R* ^2^, RMSE, and relative RMSE of 10 folds while the orange line represents the median.

The median *R* ^2^ of six ML models ranged from 0.54 (DT) to 0.7 (RF) in the validation set (Figure 3). Tree‐based models have an overall higher average *R* ^2^, smaller RMSE, and smaller rRMSE than neural networks‐based models. Within tree‐based models, RF had a higher accuracy, with a median *R* ^2^ at 0.7, an RMSE at 72.3 mg CH_4_ m^−2^ day^−1,^ and a rRMSE at 43%. In terms of neural networks‐based models, ANN has a higher accuracy, with an *R* ^2^ at 0.62, RMSE at 78.5 mg CH_4_ m^−2^ day^−1^, and rRMSE at 48%.

For the interannual variability, the median *R* ^2^ of standard deviation within a year of six ML models ranged from 0.55 (ANN) to 0.7 (RF and XGB) in the test set (Figure S2 in Supporting Information S1). Our models could well capture the seasonal cycles of the field measurements in most sites (e.g., CA‐SCB), except in some very low methane emission sites like FR‐LGT and US‐NGC (Figure S3 in Supporting Information S1).

#### 3.1.2 Methane Emission Response to Climatic and Environmental Variables

Dynamic climate variables account for 57% of feature importance on the average of 6 ML models. TAIR is the most important feature overall: the average feature importance of TAIR in the 6 ML models is 23%. As shown in Figure S4 of Supporting Information S1, except XGBoost, TAIR is assigned the highest feature importance by DT, RF, ANN, GRU, and LSTM. RH is assigned the second highest feature importance by DT and the third by RF. For the static variables, clelev and ORGC are the most important, accounting for 14.3% and 11.2% on the average of 6 ML models, respectively. clelev is the second most important in RF, ANN, GRU, and LSTM while fourth important in DT and XGB (Figure S4 in Supporting Information S1). ORGC is the third most important in ANN, GRU, and LSTM. Also, it is noted that DT and RF assign large importance to a single feature (TAIR) while XGB and NN‐based models assign the feature importance more uniformly. It indicates that all six models can capture important features (i.e., TAIR and clelev) but assign different importance to them. As a result, an ensemble of these models can avoid overemphasizing any of the features, thus leading to more accurate results.

**Figure 4:**
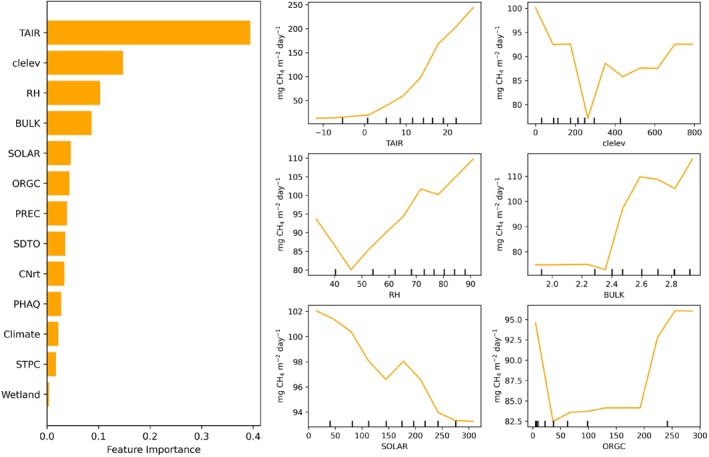
Feature importance of input variables (left panel) and partial dependent plots (right panel) of random forest model. The vertical lines of *x*‐axis represent the density of in situ data.

Also, partial dependent plots (PDPs) were used to investigate the nonlinear responses of wetland methane flux to input variables. Only the PDPs derived from the RF model are shown in Figure 4 because six ML models show similar responses and RF shows relatively better model performance. The increase in TAIR will lead to an increase in methane emissions. The response curve of methane emissions to TAIR is nearly a straight line. After exceeding 45%, methane emissions increase as the relative humidity increases. As surface downward solar radiation (SOLAR) increased, methane emissions decreased. The higher elevation (clelev) is generally associated with lower methane emissions. Methane emission increased as elevation increased after 250 m but the observed samples are relatively sparse. Soil bulk density (BULK) has less impact on methane emission until it reaches 2.23 g cm^−3^. After that, the increase in bulk will lead to an increase in methane emissions. Before reaching 40 g kg^−1^, the increase in soil organic carbon content (ORGC) leads to a decrease in methane emissions, while methane emissions will increase as ORGC becomes larger than 40 g kg^−1^.

**Figure 5:**
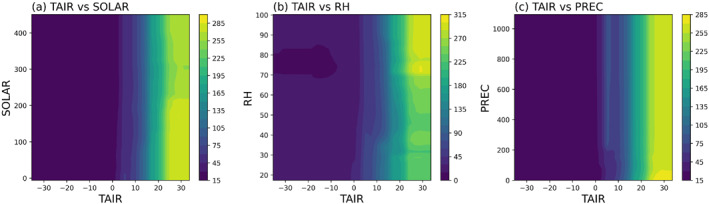
The 2‐D partial dependent plots of two variables on methane emission (unit: mg CH_4_ m^−2^ day^−1^).

To show the marginal interaction effect of dynamic variables on methane emission, we plotted the 2‐D partial dependent plots (Figure 5). As air temperature (TAIR) increased, the surface downward solar radiation (SOLAR) tended to influence methane emissions more significantly (Figure 5a). There is a notable hotspot in the plot where SOLAR is smaller than 200 W m^−2^ and (TAIR) is larger than 22°C, suggesting the optimal condition for methane emissions. Similarly, as TAIR increased, the effect of relative humidity (RH) on methane emission became more noticeable (Figure 5b). Higher relative humidity (larger than 80%) had a higher impact on methane emission when the air temperature was larger than 22°C. The interaction between air temperature (TAIR) and precipitation (PREC) (Figure 5c) was less pronounced compared to SOLAR and RH. The PREC at less than 50 mm month^−1^ and TAIR larger than 22°C could be the optimal condition for methane emission.

### 3.2 Historical Global CH_4_ Emissions

#### 3.2.1 Temporal and Spatial Variations

**Figure 6:**
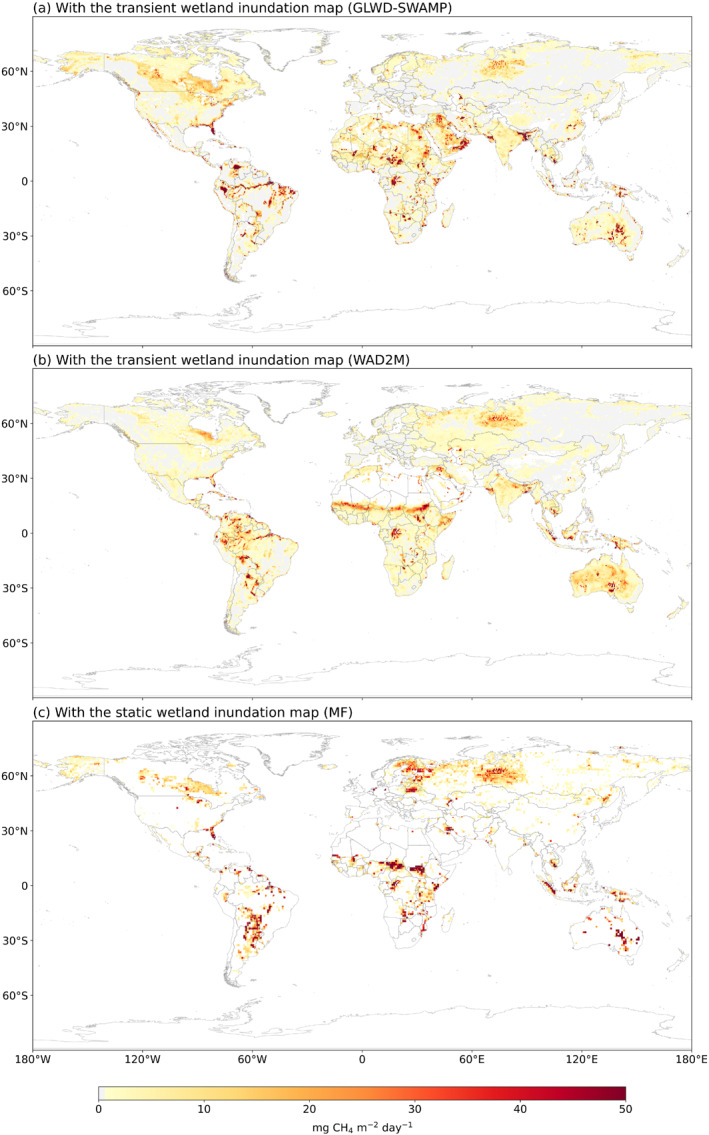
MME simulated global CH_4_ wetland emissions with the (a) GLWD‐SWAMP transient wetland inundation map from 2000 to 2012 (b) WAD2M transient wetland inundation map from 2000 to 2020 and (c) MF static wetland inundation map from 1979 to 2022.

**Figure 7:**
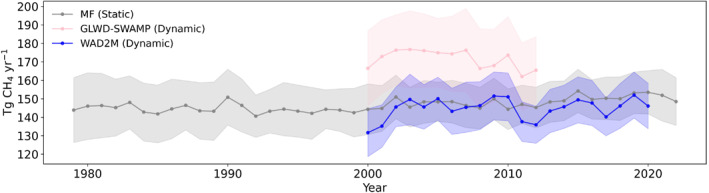
MME simulated global annual wetland emission trends with the GLWD‐SWAMP transient wetland inundation map from 2000 to 2012 (in pink), WAD2M transient wetland inundation map from 2000 to 2020 (in blue), and MF static wetland inundation map from 1979 to 2022 (in gray).

From 2000 to 2012, GLWD‐SWAMP transient wetland inundation data was used for multi‐model ensemble simulation. Areas near the equator especially the Amazon Basin, Central Africa, and the Arabian Peninsula are the hotspots for wetland CH_4_ emissions (Figure 6a). There is a noticeable increase in methane emissions from 166.5 ± 20.2 Tg CH_4_ yr^−1^ in 2000 to a peak of 176.7 ± 20.9 Tg CH_4_ yr^−1^ in 2003 (Figure 7). After reaching a peak in 2003, the trend is characterized by initial stabilization followed by fluctuating declines and a slight rebound in 2012 (Figure 7). The average global methane emission across all models is approximately 171.5 ± 19.9 Tg CH_4_ yr^−1^ (Table 1). The region of 0–30°N contributes the most emission of 75.5 ± 8 Tg CH_4_ yr^−1^, accounting for 42% of the global total wetland emissions.

**Table 1:** Historical Methane Emissions (Tg CH_4_ yr^−1^) With the ML Models From 2000 to 2012 Using the GLWD‐SWAMP Transient Wetland Inundation Map

	Global	60°S–30°S	30°S–0°	0–30°N	30°N–60°N	60°N–90°N
DT	206.1	10.9	58.7	88.1	44.5	4
RF	182.7	8.5	56.3	79.7	34.1	4
XGB	158.1	7.5	48.6	68.5	29.7	3.9
ANN	142.1	2.7	44.3	63.6	26.1	5.4
GRU	172	4.7	52.8	79.5	29.4	5.6
LSTM	168.1	3.8	51.3	73.8	31.9	7.4
Average	171.5	6.3	52	75.5	32.6	5.1
STD	19.9	2.9	4.8	8	5.8	1.3

From 2000 to 2020, WAD2M transient wetland inundation data indicated hotspots for wetland CH_4_ emissions in the near‐equatorial areas including the Amazon and Sub‐Saharan Africa (Figure 6b). It presented methane emissions increased from 131.7 ± 13.0 Tg CH_4_ yr^−1^ in 2000 to 146.1 ± 12.3 Tg CH_4_ yr^−1^ in 2020. The average global methane emission across all models is approximately 144.7 ± 11.9 Tg CH_4_ yr^−1^ (Table 2). Most methane emissions are concentrated between regions 30°S–0° and 0–30°N, with average emissions of 56.6 ± 4.9 and 55.9 ± 4.3 Tg CH_4_ yr^−1^, respectively.

**Table 2:** Historical Methane Emissions (Tg CH_4_ yr^−1^) With the ML Models From 2000 to 2020 Using the WAD2M Transient Wetland Inundation Map

	Global	60°S–30°S	30°S–0°	0–30°N	30°N–60°N	60°N–90°N
DT	159.4	10.4	61.7	57	27.2	3.1
RF	157.7	8.7	61.4	60.2	24.3	3.1
XGB	132.7	7.7	51.4	50.5	20.3	2.8
ANN	129.4	2.8	49.7	54.3	18.3	4.3
GRU	151.3	4.5	60.8	62.1	19.4	4.5
LSTM	138.1	3.4	54.5	51.4	22.1	6.6
Average	144.7	6.2	56.6	55.9	21.9	4.1
STD	11.9	2.8	4.9	4.3	3.1	1.3

From 1979 to 2022, MF static wetland map was used for multi‐model ensemble simulation. Northern Europe and near‐equatorial areas (e.g., the center of South America and central Africa) are the hotspots for wetland methane emissions (Figure 6c). From an initial level of approximately 143.9 ± 17.6 Tg CH_4_ yr^−1^ in 1979, the emissions appear to trend upward to 148.5 ± 12.9 Tg CH_4_ yr^−1^ in 2022, albeit with some variabilities (Figure 7). The peak value is 154.3 ± 11.7 Tg CH_4_ yr^−1^ (2015), and the trough value is 140.6 ± 11.5 Tg CH_4_ yr^−1^ (1992). The average global methane emission across all models is 146.6 ± 12.2 Tg CH_4_ yr^−1^ (Table 3). Most methane emissions appear to be concentrated between latitudes 30°S–0° and 0–30°N, with average emissions of 53.7 ± 4 and 49.1 ± 4.1 Tg CH_4_ yr^−1^, respectively. This suggests that equatorial regions might play significant contributors to global methane emissions. On the other hand, the latitudinal range 60°N–90°N shows the lowest average methane emissions at 13.4 ± 3.3 Tg CH_4_ yr^−1^, suggesting less methane‐producing activity in these regions.

**Table 3:** Historical Methane Emissions (Tg CH_4_ yr^−1^) Estimated With the ML Models During the Period 1979–2022 Using MF Wetland Area

	Global	60°S–30°S	30°S–0°	0–30°N	30°N–60°N	60°N–90°N
DT	160.1	9.3	57.7	54.7	26.8	11.5
RF	155.4	8.5	58.3	53.1	24.6	10.9
XGB	134.8	7.4	50.8	46.1	20.7	9.8
ANN	126.5	2.5	46.8	42.8	20.5	13.9
GRU	147.2	3.9	55.6	50.7	22.4	14.7
LSTM	155.7	3.3	52.8	47.1	32.6	19.9
Average	146.6	5.8	53.7	49.1	24.6	13.4
STD	12.2	2.7	4	4.1	4.2	3.3

Overall, there was a larger ML model uncertainty in the tropical regions (30°N–30°S) while smaller uncertainties in temperate regions (>30°N) (Figure S5 in Supporting Information S1). Wetland CH_4_ emissions estimated by the WAD2M transient wetland inundation map and MF static wetland map presented a similar spatial distribution except in Northern Europe (Figures 6b and 6c). They are also close for the perspective of the global annual average: 144.7 ± 11.9 Tg CH_4_ yr^−1^ for WAD2M maps and 146.6 ± 12.2 Tg CH_4_ yr^−1^ for MF maps. However, the GLWD‐SWAMP transient wetland inundation map estimated a higher annual average CH_4_ emissions (171.5 ± 19.9 Tg CH_4_ yr^−1^). Especially in region 0–30°N, models driven by GLWD‐SWAMPS maps show substantially higher average emissions of 75.5 ± 8 Tg CH_4_ yr^−1^, as opposed to 55.9 ± 4.3 Tg CH_4_ yr^−1^ in models using WAD2M wetland distribution maps and 49.1 ± 4.1 Tg CH_4_ yr^−1^ in models using MF wetland distribution maps. This is because GLWD‐SWAMPS indicated a large wetland proportion in the Sahara region while WAD2M and MF do not.

If using MF static wetland maps for future projection, the biases caused by this assumption are −3.7 ± 0.7 Tg CH_4_ yr^−1^ compared with WAD2M wetland maps and 24.7 ± 8.5 Tg CH_4_ yr^−1^ compared with GLWD‐SWAMP wetland maps.

#### 3.2.2 Seasonality

**Figure 8:**
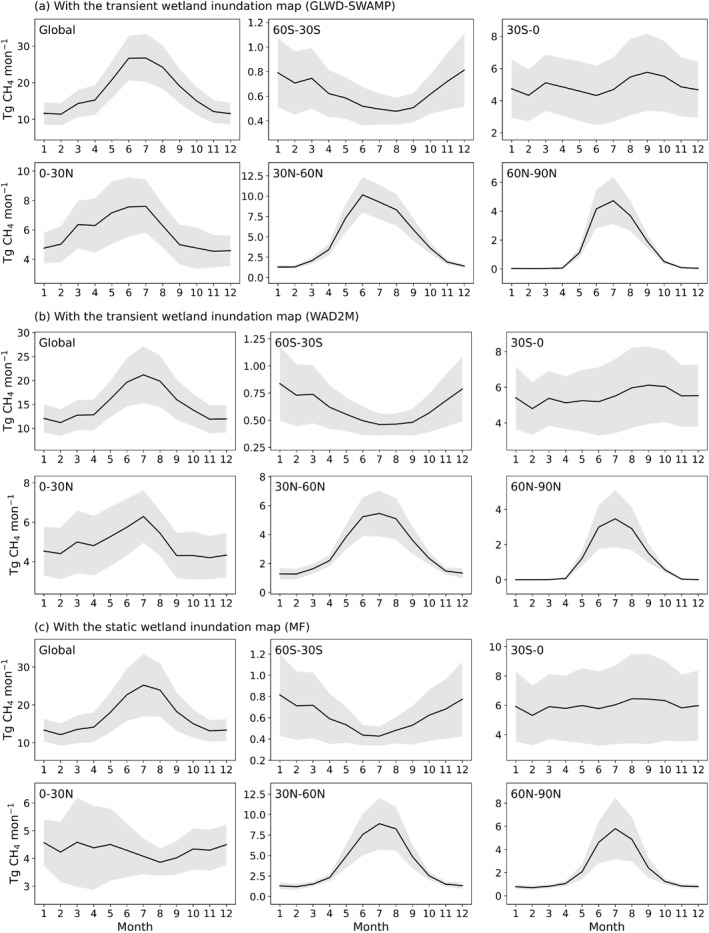
The mean seasonal cycle of model‐predicted CH_4_ flux (Tg CH_4_ month^−1^) with the (a) GLWD‐SWAMP transient wetland inundation map from 2000 to 2012, (b) WAD2M transient wetland inundation map from 2000 to 2020 and (c) MF static wetland inundation map from 1979 to 2022. The solid black line represents the average of different ML models. The gray shadow represents the standard deviations.

The wetland methane emissions using transient and static wetland maps show generally similar seasonality (Figure 8). Global CH_4_ emissions are higher during JJA (June, July, and August; summer) and lower during DJF (December, January, and February; winter) and have a higher uncertainty during JJA. The global peak methane emission that occurred in the JJA at the global scale mainly corresponds with the warmer soil temperatures and peak productivity at northern high latitudes. From a regional perspective, the 60°S–30°S region has the opposite seasonality compared to global results. The 30°S–0 region has a two‐peak shape. The 30°N–60°N and 60°N–90°N regions are very similar to global results, with a peak at JJA. The wetland methane emissions using transient and static wetland maps show differences in the region 0–30°N. The wetland methane emissions using the transient wetland map show a peak in the JJA while the wetland methane emissions using the static wetland map show a peak in the MAM (March, April, and May; Spring).

### 3.3 Upscaling Future Global Methane Emissions

#### 3.3.1 Spatial Distribution of CH_4_ Emissions

**Figure 9:**
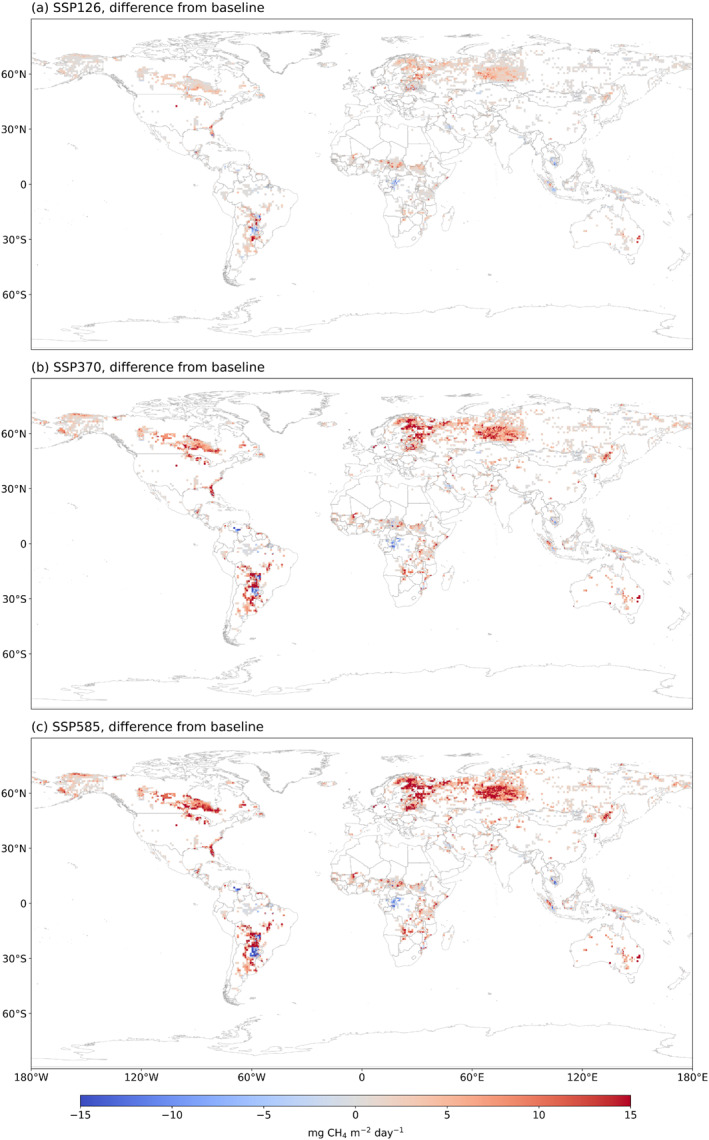
Multi‐model ensemble (MME) future predictions of global mean wetland CH_4_ emission distribution during 2081–2099 comparing with baseline estimates during 1995–2014.

The methane emissions will reach 165.8 ± 11.6, 185.6 ± 15.0, and 193.6 ± 17.2 Tg CH_4_ yr^−1^ during the last two decades of the 21st century under SSP126, SSP370, and SSP 585 scenarios, respectively. Our model simulations during 2080–2099 under SSP126, SSP370, and SSP585 were compared with a baseline simulation (1995–2014) (Figure 9). With the existing high methane emissions in the tropical area, the emission in the arctic and temperate areas will significantly increase in the future largely due to temperature increase‐induced melting of ice and snow (Figure 9). Under the SSP126 scenario (Figure 9a), emission increases will be primarily from the La Plata Basin in South America. Under the SSP370 scenario, the increase in methane emission will be larger in the La Plata Basin and the increased areas will expand to western Siberia, Canadian lowlands, and Northern Europe (Figure 9b). Under the SSP585, the emissions from wetlands in western Siberia and Canadian lowlands may increase by more than 15 mg CH_4_ m^−2^ day^−1^ (Figure 9c).

#### 3.3.2 Temporal Trends of CH_4_ Dynamics

**Figure 10:**
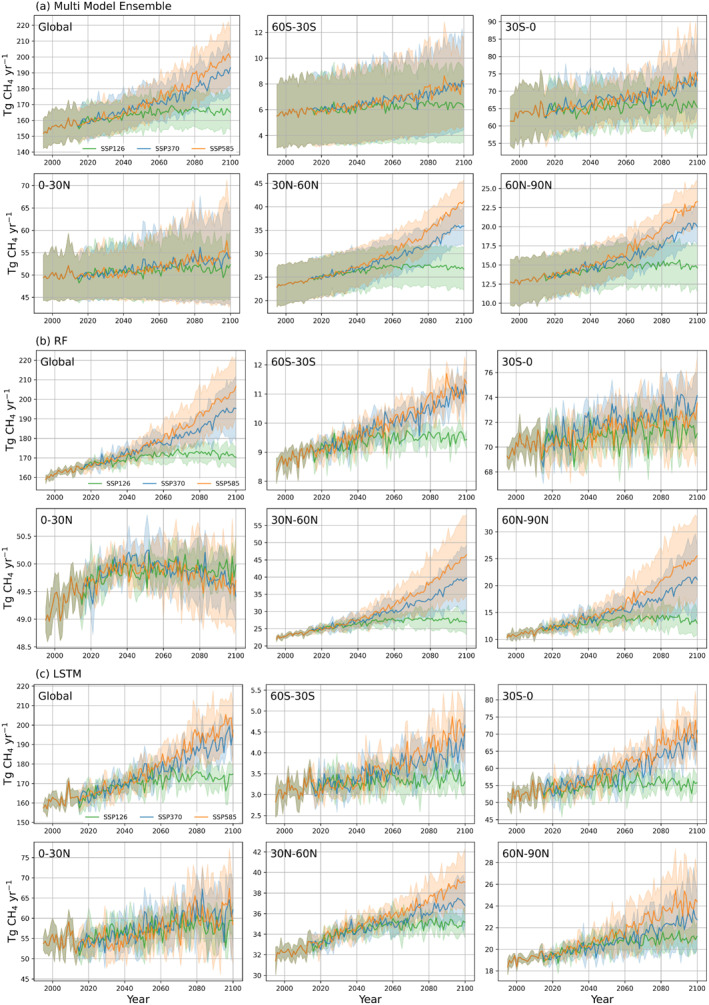
Model future predictions of annual wetland emissions of different regions: The solid lines represent the average methane emissions driven by 5 GCMs. The shaded areas represent the 1*σ* range of the estimates from 5 GCMs. The green, blue, and orange colors represent model predictions driven by SSP126, SSP370, and SSP585.

Mean global annual CH_4_ emissions from natural wetlands were projected to increase from 152 ± 9.5 Tg CH_4_ yr⁻^1^ in 1995 to 165 ± 11.2 Tg CH_4_ yr⁻^1^ (SSP126), 193 ± 17.3 Tg CH_4_ yr⁻^1^ (SSP370), and 200 ± 16.7 Tg CH_4_ yr⁻^1^ (SSP585) by 2100 (Figure 10). In the sustainability scenario (SSP126 scenario), which aims to keep the radiative forcing to 2.6 W/m^2^ by the year 2100, wetland CH_4_ emissions are projected to peak around the 2060s, with an average of ∼167 Tg CH_4_ yr^−1^, and stay stable thereafter (Figure 10a). In the regional rivalry scenario (SSP370), where countries emphasize energy security and access to resources rather than environmental concerns, methane emissions will increase to ∼190 Tg CH_4_ yr^−1^ by the end of the century. In the fossil‐fueled development Scenario (SSP585), where the global economy grows rapidly and emphasis on fossil fuel use, the increase of methane emission is significant, reaching 200 Tg CH_4_ yr^−1^ by the end of this century (Figure 10a).

In the future, methane emissions are projected to increase globally, with the hotspots in the temperate and arctic regions and scatter distribution in South America (Figure S6 in Supporting Information S1). Regions 30°S–0° and 0–30°N contributed the most to the global methane emission (Figure 10a). The region of 30°S–0° increased significantly under the SSP370 and SSP585 scenarios by the 2090s, which was agreed upon by most ML models. However, there are some disagreements for 0–30°N. RF predicted methane emissions will increase by 1 Tg CH_4_ yr^−1^ and then slightly decrease by 0.5 Tg CH_4_ yr^−1^ (Figure 10b). However, LSTM predicted an increasing trend of methane emission in the region 0–30°N (Figure 10c). The uncertainty of using ML models is slightly larger than using different GCM climate forcing (Figures S7 and S8 in Supporting Information S1). The hotspots with larger ML model uncertainty are the West of the Ural Mountains, South America, and Africa (Figure S7 in Supporting Information S1) while the hotspots with larger GCM uncertainty are South America and the West of the Ural Mountains (Figure S8 in Supporting Information S1).

## 4 Discussion

### 4.1 Performance of Various Machine Learning Models

In terms of individual model performance, RF and XGB have slightly higher accuracies than NN models. This is due to the training samples being insufficient to well train the NN models in all regions. In terms of NN models, ANN model has slightly better performance than the GRU and LSTM since both of them are designed for the time‐series feature variables while the input feature is not time series in this study. Therefore, due to the coarse temporal resolution of the climate features, the performance of GRU and LSTM is limited. Also, models have predicted different magnitudes of global annual mean methane emission. Specifically, ANN gave the lowest emission estimates at 126.5 Tg CH_4_ yr^−1^ during 1979–2022 while DT gave the highest estimation at 160.1 Tg CH_4_ yr^−1^ (Table 3). We also examined the variations in the estimated trends of methane emissions in 2000–2099 by each model (Figure 6). These models have similar predictions regarding the trends of global methane emissions. The high agreement is also observed in regions including northern mid‐ and high‐latitude regions. However, some disagreement occurs in the equatorial regions. For example, RF predicted a stagnation pattern while LSTM predicted an increasing trend of methane emission in 0–30°N (Figure 10).

NN models show overall lower emissions than TB models. This is because most of the training samples are from boreal regions, which corresponds to lower methane emissions compared to the other regions. Those samples would contribute more to the model training. NN models are characterized by inherent complexity and the capacity to capture intricate patterns in big data. The limited availability of methane observation may limit the generalization of NN models from boreal regions to tropical regions. Thus, NN tends to give lower‐than‐average emission estimation. To balance the advantages and disadvantages of different models, we combined these six machine learning models to develop the MME model estimates in this research. Future direction can include the process‐based model generated synthetic data as training samples to enrich the data set for NN models to fully leverage the power of NN (L. Liu et al., 2022; Ma et al., 2024).

### 4.2 Effects of Input Variables on the Inventory

The spatial and temporal extent of anoxia, temperature, and substrate availability are the most essential factors that contribute to wetland methane production (Saunois et al., 2020, pp. 2000–2017; Valentine et al., 1994; Wania et al., 2010; Whalen, 2005). The related variables presented high importance in our model training process (Figure 4 left panel).

Among all variables, climate variables, especially temperature, are the most important input features (Figure 4 left panel). Methanogens predominantly exhibit mesophilic characteristics, typically favoring a temperature range of 30–40°C for optimal growth (Whalen, 2005; Zinder, 1993). The higher temperature can accelerate methane emission by enhancing microbial activity and the decomposition of organic matter in wetlands because methane is primarily produced as a byproduct of anaerobic decomposition in wetlands. Precipitation has nonlinear effects on methane emissions (Turetsky et al., 2014). The increase in precipitation enhances the methane emissions from wetlands as it increases the formation of waterlogged soils, creating anoxic conditions for methanogens. However, excessive rainfall could constrain the methane flux because the diffusion through water will be constrained. At the same time there may be also a decrease in plant carbon inputs to soils due to flooding or enhanced availability of oxygen and other electron acceptors in flowing water (Turetsky et al., 2014). But at the same time, temperature also plays a role and causes contrasting effects. Higher temperatures can enhance gross primary productivity and lead to greater carbon allocation to root systems belowground as well as improve methane diffusion. Higher solar radiation can promote plant growth, which is a source of organic matter. When they are decomposed by microbes in anaerobic conditions, they will produce methane as a byproduct. However, beyond a certain threshold, the higher solar radiation may be associated with dry months when the water table drops and then reduces the methane emission.

Topography and soil also have a large impact on methane emissions (Figure 4 left panel). Higher elevations often have better drainage which can lead to drier soil conditions, and in turn, can inhibit the activity of methanogenic microbes that produce methane in waterlogged anaerobic conditions. For the soil properties, higher organic carbon content in the soil potentially leads to an increase in methane emission because it can provide more substrate for the microbes. Previous research found that steady‐state fermentation and methanogenesis were proportional to peat carbon, suggesting soil carbon is a strong predictor for methane production under long‐term anaerobic conditions (Blodau, 2011; Bonaiuti et al., 2017, 2020).

### 4.3 Effects of Various Wetland Distribution Data on the Inventory

The estimation of regional CH_4_ emissions may be greatly affected by the choice of wetland data set being used. The use of GLWD‐SWAMPS, WAD2M, and MF wetland distribution maps resulted in different regional distributions of methane fluxes (Figure 6). MF static wetland map indicates a larger area of high wetland fraction in the West of the Ural Mountains than GLWD‐SWAMPS and WAD2M (Figure S1 in Supporting Information S1). GLWD‐SWAMPS presented a larger wetland fraction in the Saharan regions than MF and WAD2M. MF static wetland map is overall closer to WAD2M than GLWD‐SWAMPS. Also, GLWD‐SWAMPS showed an increasing trend while MF showed a decreasing trend (Figure 7). This is because the GLWD‐SWAMPS considers the impact of changes in wetland area on methane emissions, while the MF assumes that the wetland area remains unchanged and only considers the impact of climate change. This finding was consistent with a previous study, which suggested that the increasing global wetland CH_4_ emissions were mainly related to temperature and changing global wetland area (Z. Zhang et al., 2017).

It is worth noting that the future projection is based on a static wetland map and may not capture the impacts of global hydrological change on wetland distribution, especially in tropical/monsoon regions. The biases caused by the static wetland assumption are −3.7 ± 0.7 Tg CH_4_ yr^−1^ compared with WAD2M wetland maps and 24.7 ± 8.5 Tg CH_4_ yr^−1^ compared with GLWD‐SWAMP wetland maps. WAD2M could be a better reference to quantify the biases caused by the static wetland map assumptions than GLWD‐SWAMP. WAD2M is developed based on GLWD‐SWAMP. Compared with GLWD‐SWAMP, (a) WAD2M used the updated inundated area fraction derived from microwave remote sensing data set, SWAMPS v3.2; (b) WAD2M used multiple static wetland maps as mergers while SWAMPS‐GWLD only used GLWD; (c) WAD2M removed the lakes, ponds, rivers, streams, and irrigated rice paddies and used a globally consistent ocean land mask (Z. Zhang et al., 2021).

### 4.4 Comparisons With Existing Estimates

Global wetland CH_4_ emissions and overall uncertainties of our MME simulation were 171.5 ± 19.9 Tg CH_4_ yr^−1^ during 2000–2012 using GLWD‐SWAMP transient wetland area, 144.7 ± 11.9 Tg CH_4_ yr^−1^ during 2000–2020 using WAD2M V2 transient wetland map, and 146.6 ± 12.2 Tg CH_4_ yr^−1^ during 1979–2022 using MF static wetland area. Our result (144.7 ± 11.9 Tg CH_4_ yr^−1^, WAD2M) agrees with the only current ML‐based global upscaling product (McNicol et al., 2023), which was generated from the random forest model and reported an average of 146 ± 42.7 Tg CH_4_ yr^−1^ using climate variables from MERRA‐2 reanalysis, remote sensing data, and WAD2M wetland area (McNicol et al., 2023). Our GLWD‐SWAMP results (171.5 ± 19.9 Tg CH_4_ yr^−1^) are close to our past estimates using the process‐based Terrestrial Ecosystem Model (TEM) model, which indicated that the global wetland emissions ranged from 186 to 212 CH_4_ year^−1^ during 2000–2012 (L. Liu et al., 2020). Compared with some other studies, our results also fall within the given range. For example, recent intra‐model comparison studies reported a mean value of 183 Tg CH_4_ yr^−1^ with a range of 151–222 Tg CH_4_ yr^−1^ from 2000 to 2012 (Saunois et al., 2016) and a range of 102–200 Tg CH_4_ yr^−1^ from 2000 to 2017 (Saunois et al., 2020). Kirschke et al. reported a mean value of 217 Tg CH_4_ yr^−1^ with a range of 177–284 Tg CH_4_ yr^−1^, which is slightly higher than ours (Kirschke et al., 2013).

Spatially, our results suggested that the tropics are a major emission source (Figure 6). This is consistent with previous findings from inversion and observational studies in the Amazon Basin, indicating the region is a substantial source of methane (Devol et al., 1988; Pangala et al., 2017). Specifically, our estimates are 118.7 Tg CH_4_ yr^−1^ in 60°S–30°N from 2000 to 2020 using the WAD2M V2 map and 108.6 Tg CH_4_ yr^−1^ from 1979 to 2022 using the MF map. Comparatively, the methane emissions presented in previous studies were 118 Tg CH_4_ yr^−1^ (McNicol et al., 2023), which is close to our estimates. Saunois et al. estimated that 71–155 Tg CH_4_ yr^−1^ in 30°S–0°, 11–44 Tg CH_4_ yr^−1^ in 30°N–60°N and 2–18 Tg CH_4_ yr^−1^ in 60°N–90°N (Saunois et al., 2020). Our estimates fall within the ranges (Tables 1, 2, 3).

Our MME projected that wetland methane emission would increase by 13.1% (SSP126), 26.6% (SSP370), and 32.0% (SSP585) by 2080–2099. It was projected to increase from 152 ± 9.5 Tg CH_4_ yr⁻^1^ in 1995 to 165 ± 11.2 Tg CH_4_ yr⁻^1^ (SSP126), 193 ± 17.3 Tg CH_4_ yr⁻^1^ (SSP370), and 200 ± 16.7 Tg CH_4_ yr⁻^1^ (SSP585) by 2100 (Figure 10). The existing future projections based on process‐based models are larger than our projections. Shindell et al. (2013) used the GISS climate model (GISS—E2) in support of Coupled Model Intercomparison Project Phase 5 (CMIP5) and projected future wetland methane will increase by 20% under RCP 8.5 until 2100 (Shindell et al., 2013). Specifically, they predicted that it will increase from around 195 Tg CH_4_ yr⁻^1^ in 2005 to 202 (RCP 2.6) and 236 (RCP 8.5) Tg CH_4_ yr⁻^1^ in 2100. Koffi et al. (2020) developed an atmospheric inverse model of CH_4_ fluxes based on observed temperature and precipitation and then projected current emissions will increase from 175 Tg CH_4_ yr⁻^1^ to 190 (RCP2.6) and 290 (RCP8.5) Tg CH_4_ yr⁻^1^ in 2100 using CMIP5 climate forcing (Koffi et al., 2020). Its estimates of RCP2.6 are similar to our result, but there is a significant difference in the estimates of RCP8.5 (238 ± 27 vs. 290 Tg CH_4_ yr⁻^1^). Z. Zhang et al. (2017) used LPJ‐wsl driven by CMIP5 climate forcing and show the mean global annual methane emissions from natural wetlands will increase from 172 ± 12 Tg CH_4_ yr⁻^1^ in 2000 to 221.6 ± 15 (RCP2.6), and 338 ± 28 Tg CH_4_ yr⁻^1^ (RCP8.5) in 2100 (Z. Zhang et al., 2017, 2023), which are overall higher than our results and there are also large differences in RCP8.5. They used a larger wetland distribution map and considered the change of wetland area over time, which could explain the higher values. It is worth mentioning that another recent study using MPI‐ESM and CMIP6 projected around 280 (SSP126), 450 (SSP370), and 500 (SSP585) Tg CH_4_ yr⁻^1^ in 2100, which is significantly larger than all the above projections (Kleinen et al., 2021).

### 4.5 Uncertainty and Limitations

Some uncertainties and limitations in this research need to be considered. First, wetland area maps can be the major uncertainty source, as also identified in previous studies (L. Liu et al., 2020; McNicol et al., 2023; Melton et al., 2013; Z. Zhang et al., 2017). Minor inaccuracies in the determination of wetland areas and their classification can result in considerable disparities in the estimation of methane flux at the regional level (Tian et al., 2015). Second, input variables derived from different general climate models (GCMs) also introduce uncertainty. This study finds that the uncertainty originating from various GCMs is relatively minimal during historical periods but tends to increase over time (Figures 10b and 10c). Third, the uncertainties can be from the ML models. The uncertainty associated with different ML models is found to be greater than that of GCMs in this study (Figures S7 and S8 in Supporting Information S1). The model uncertainties can be attributed to the different model structures and the black‐box nature of current ML approaches, which causes large uncertainty in extrapolation or out‐of‐sample projections (Hutson, 2022). Future direction can be incorporating scientific knowledge into the ML models to guide the projection (Irrgang et al., 2021; Reichstein et al., 2019; Willard et al., 2022). Fourthly, WISE30sec soil properties database used in the study is generated from ∼21,000 soil profiles and designed for broad‐scale modeling (Batjes, 2016). Although it was widely used as reference data in wetland‐related literature (Qiu et al., 2019), wetland soils are often minority cover types at the landscape scale. Using wetland‐specific soil properties databases will further improve the accuracy of our future research. Lastly, the limitation of in situ data also introduced uncertainties. Methane emissions from tropical areas contribute significantly to the global sum. Although the sites used in this research cover the sites used in McNicol et al. (2023), data available from these regions are considerably less abundant than from other regions. This imbalance in data availability, coupled with insufficient data for training machine learning models, hampers the overall performance and accuracy of these models.

## 5 Conclusions

This study quantifies the global wetland methane emissions in contemporary and future periods by integrating a machine learning‐based MME approach, wetland CH_4_ flux measurements from both chamber and EC flux tower, and their associated environmental data. We find that global mean annual wetland CH_4_ emissions are 171.5 Tg CH_4_ yr^−1^ with an uncertainty range of 142.1–206.1 Tg CH_4_ yr^−1^ during 2000–2012 using the transient wetland inundation data. By using the static wetland distribution data, the emissions are estimated to be 146.6 ± 12.2 Tg CH_4_ yr^−1^ during 1979–2022 but have large differences in regional distributions. The estimated emissions will reach 165.8 ± 11.6, 185.6 ± 15.0, and 193.6 ± 17.2 Tg CH_4_ yr^−1^ in the last two decades of the 21st century when using the SSP126, SSP370, and SSP585, respectively. Among the six models tested, RF has the best performance in the validation set, with an RMSE at 72.3 mg CH_4_ m^−2^ day^−1^. In contrast, DT has the lowest accuracy, with an RMSE of 90.1 mg CH_4_ m^−2^ day^−1^. Northern Europe and near‐equatorial areas are the hotspots of wetland methane emissions. The global wetland CH_4_ emissions are most sensitive to changes in temperature, soil organic matter, elevation, and relative humidity. This study has provided valuable insights into methane emission from a data‐driven perspective and called for the need for comprehensive CH_4_ measurements, especially in arid and tropical regions, and better characterization of variations of wetlands to improve future inventorying of wetland CH_4_ emissions at the global scale.

## Supporting information

Supporting Information S1

## Data Availability

The ERA5 climate data are available in Hersbach et al. (2023). The ISIMIP3b climate data are available in Lange and Büchner (2021). The site‐level CH_4_ flux data obtained from 35 chamber sites and 47 eddy covariance sites are available in S. Chen et al. (2024b). The generated CH_4_ emissions data are available in S. Chen et al. (2024a).
