# Supplementary material for: Quantifying Global Wetland Methane Emissions With In Situ Methane Flux Data and Machine Learning Approaches
Source: Earths Future. 2024 Oct 31;12(11):e2023EF004330. doi: 10.1029/2023EF004330 (PMC11607141; doi:10.1029/2023EF004330)
Supplement: Supplementary file 1 — Supporting Information S1 [file EFT2-12-0-s001.pdf]

AGU PUBLICATIONS

Earth's Future

Supporting Information for

Quantifying Global Wetland Methane Emissions with In Situ Methane Flux Data and Machine Learning Approaches

Shuo Chen[1], Licheng Liu[1], Yuchi Ma[2], Qianlai Zhuang[1,3], Narasinha J. Shurpali[4]

[1]Department of Earth, Atmospheric, Planetary Sciences, Purdue University, West Lafayette, IN 47907, USA
[2]Department of Earth System Science and Center on Food Security and the Environment, Stanford University, Stanford, CA, 94305, USA
[3]Department of Agronomy, Purdue University, West Lafayette, IN 47907, USA
[4]Production Systems Unit, Natural Resources Institute Finland (Luke), Halolantie 31A, 71750, Maaninka Finland

Correspondence to: qzhuang@purdue.edu

Contents of this file

Figures S1 to S8
Tables S1 to S4

Introduction

The method used to generate the following supporting information is described in *Quantifying Global Wetland Methane Emissions with In Situ Methane Flux Data and Machine Learning Approaches*.

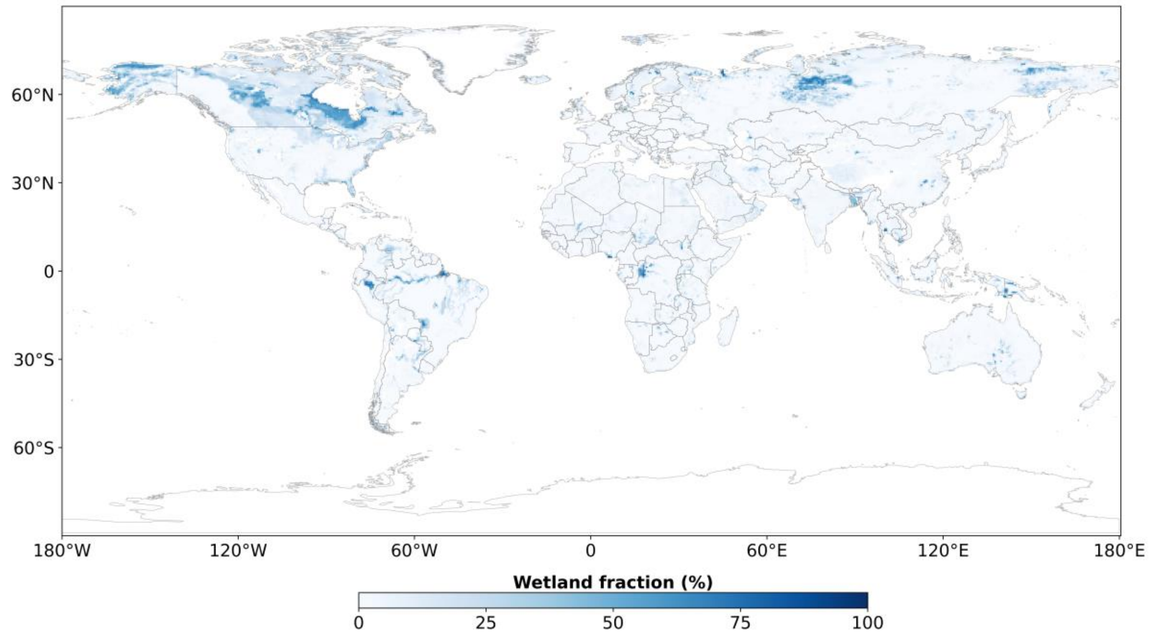

Figure S1. GLWD-SWAMP wetland fraction

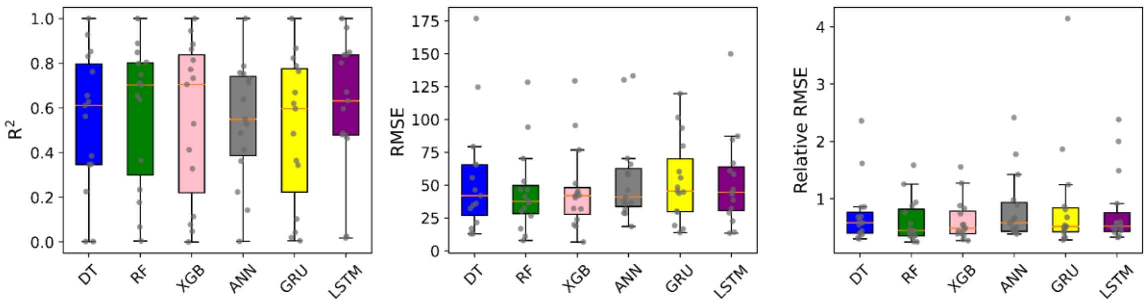

Figure S2. The comparisons of prediction accuracy for the standard deviation of methane emissions from leave-one-year-out cross-validation.

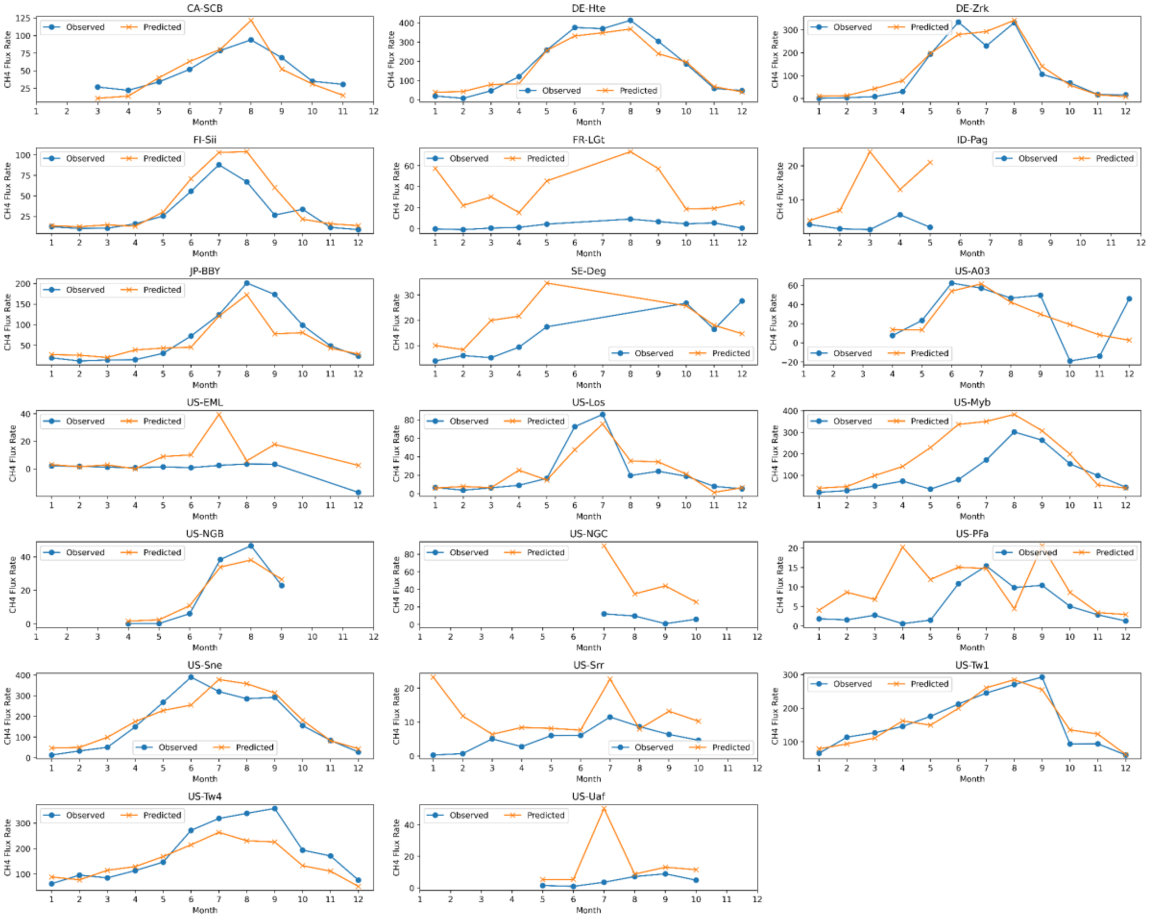

Figure S3. Seasonal cycles of methane emissions in 2017

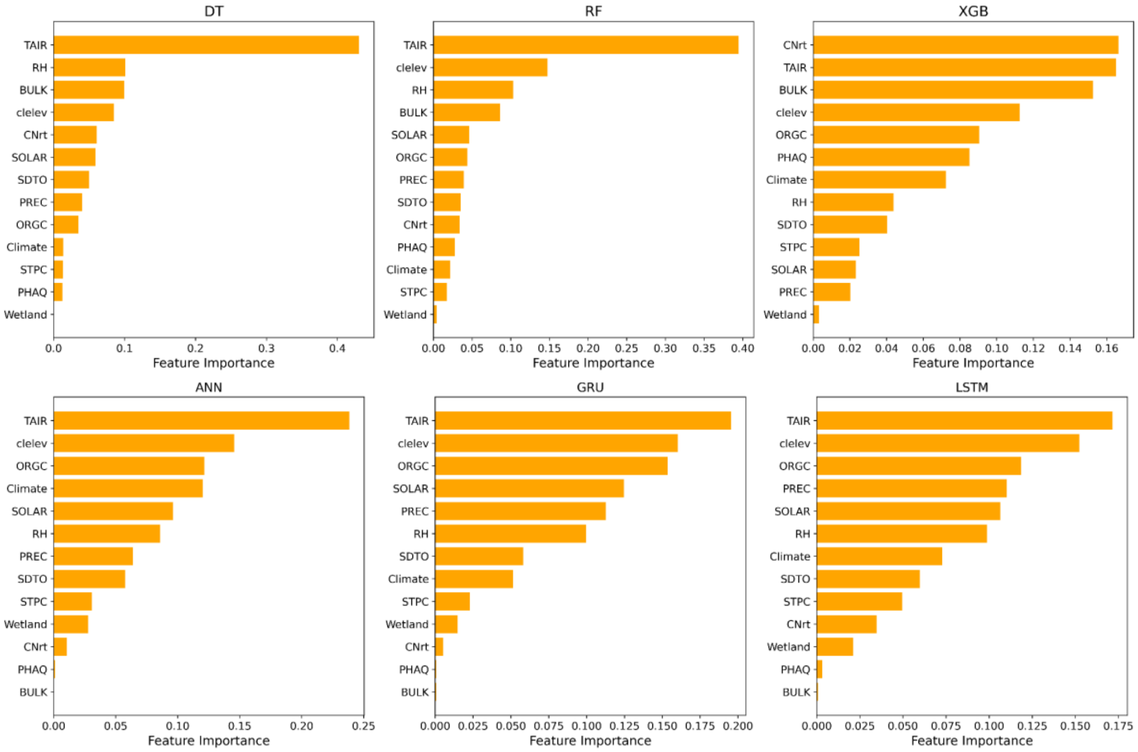

Figure S4. Feature importance of 6 machine learning models

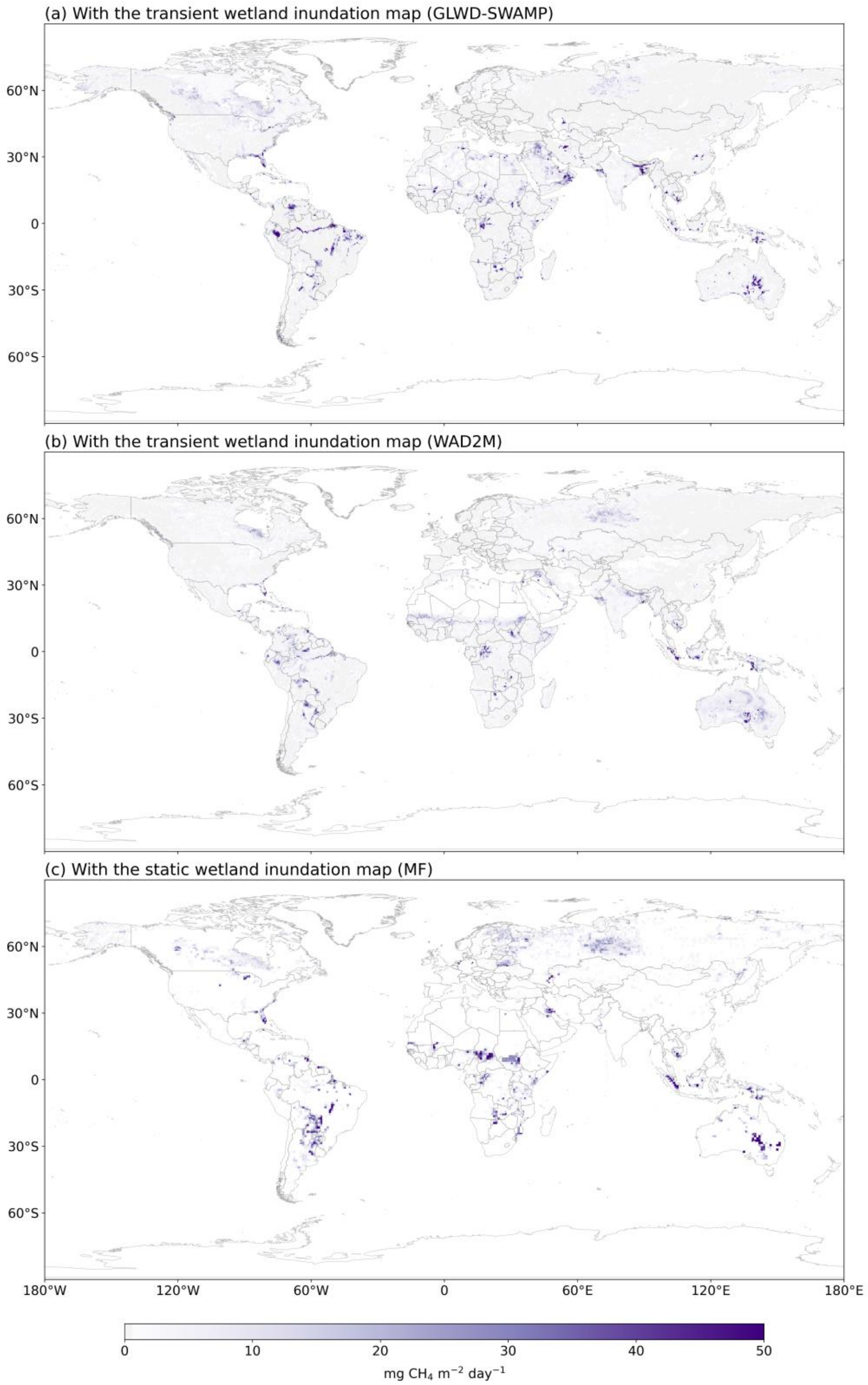

Figure S5. Uncertainty of ML models in simulating historical wetland methane emissions with the (a) GLWD-SWAMP transient wetland inundation map from 2000 to 2012 (b) WAD2M transient wetland inundation map from 2000 to 2020 and (c) MF static wetland inundation map from 1979 to 2022.

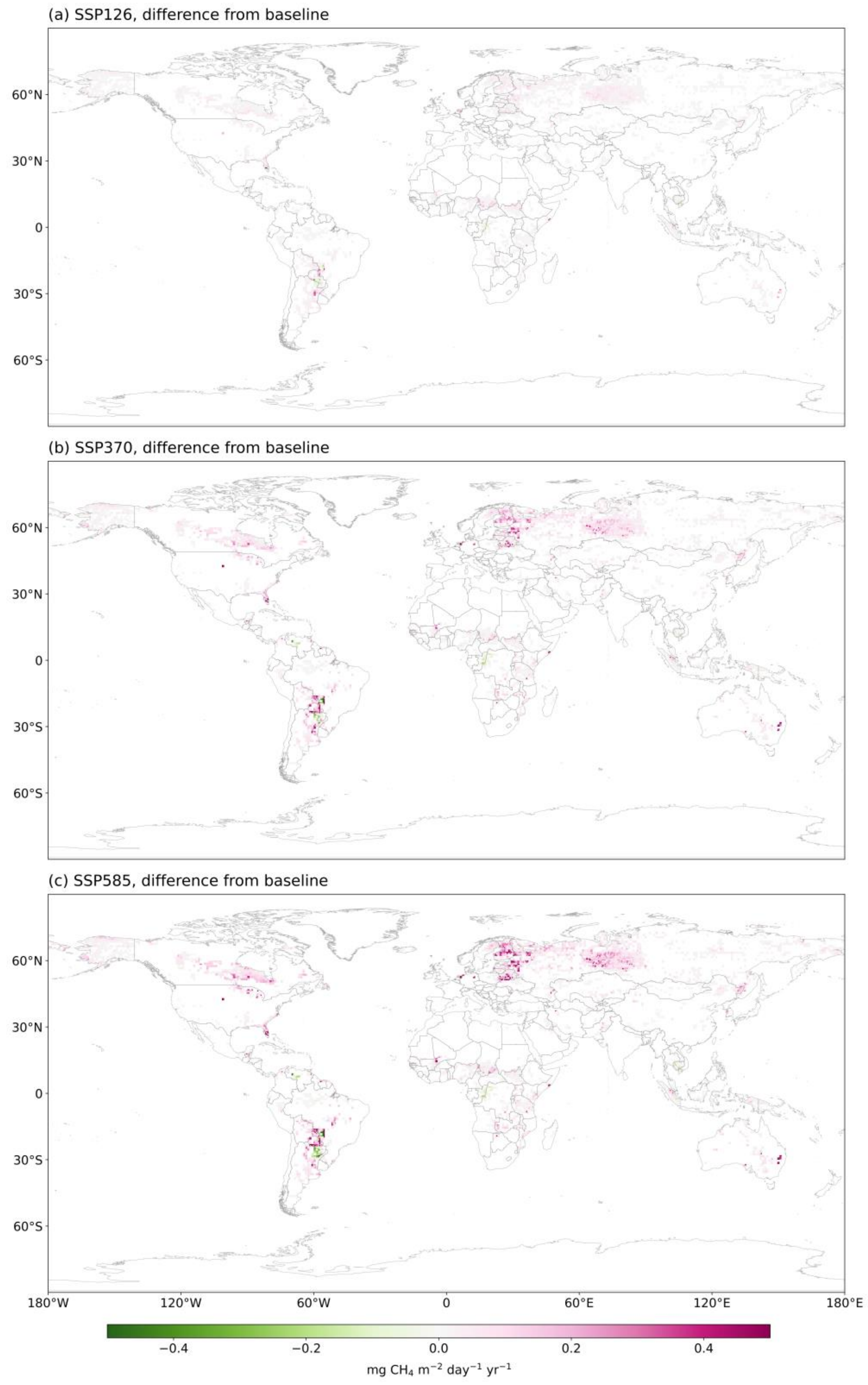

Figure S6. Future trends of global wetland methane emissions from 1995 to 2100 under SSP126, SSP370, and SSP585 simulated by multi-model ensemble (MME).

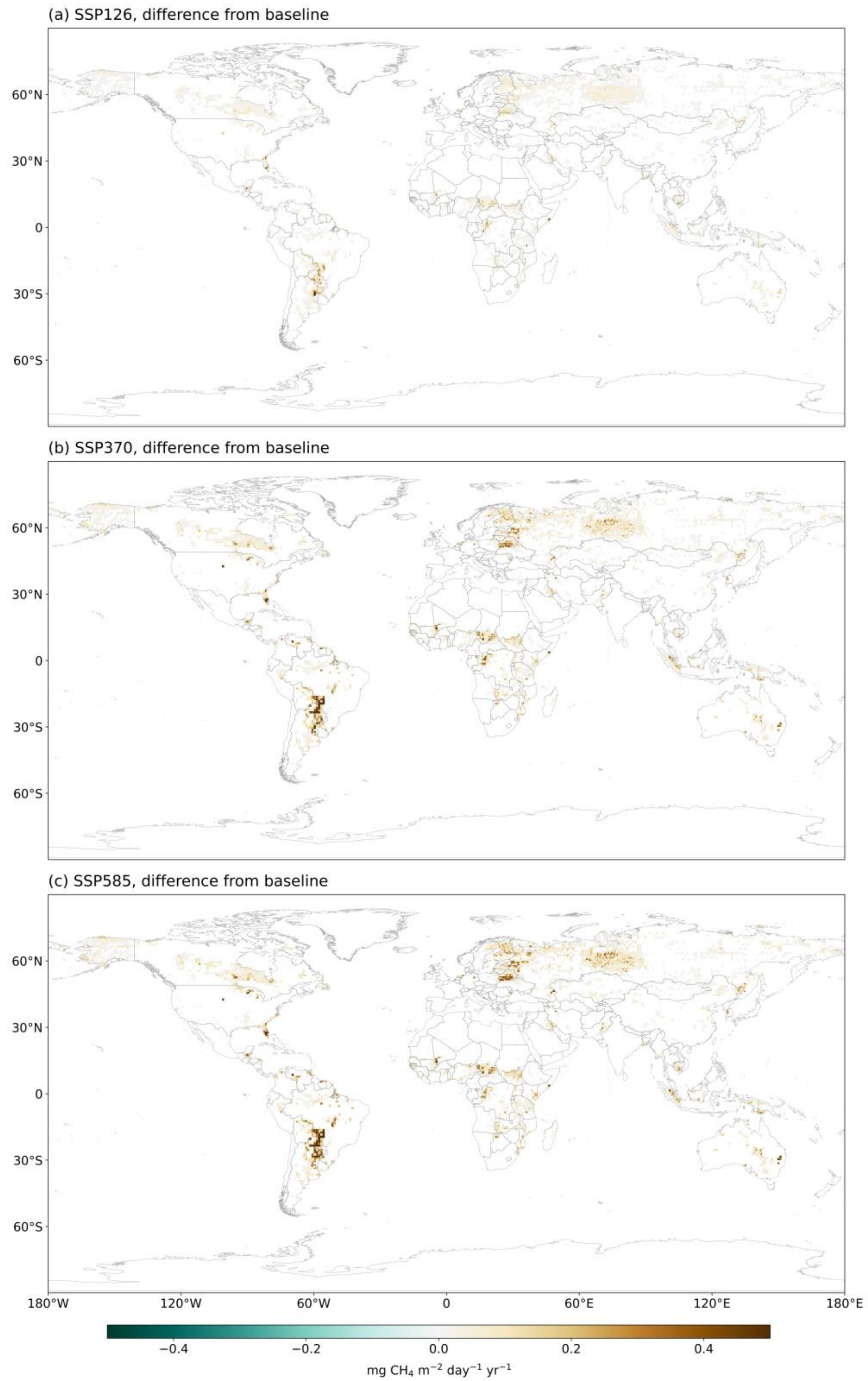

Figure S7. Uncertainty of ML models in simulating future trends of global wetland methane emissions from 1995 to 2100 under SSP126, SSP370, and SSP585.

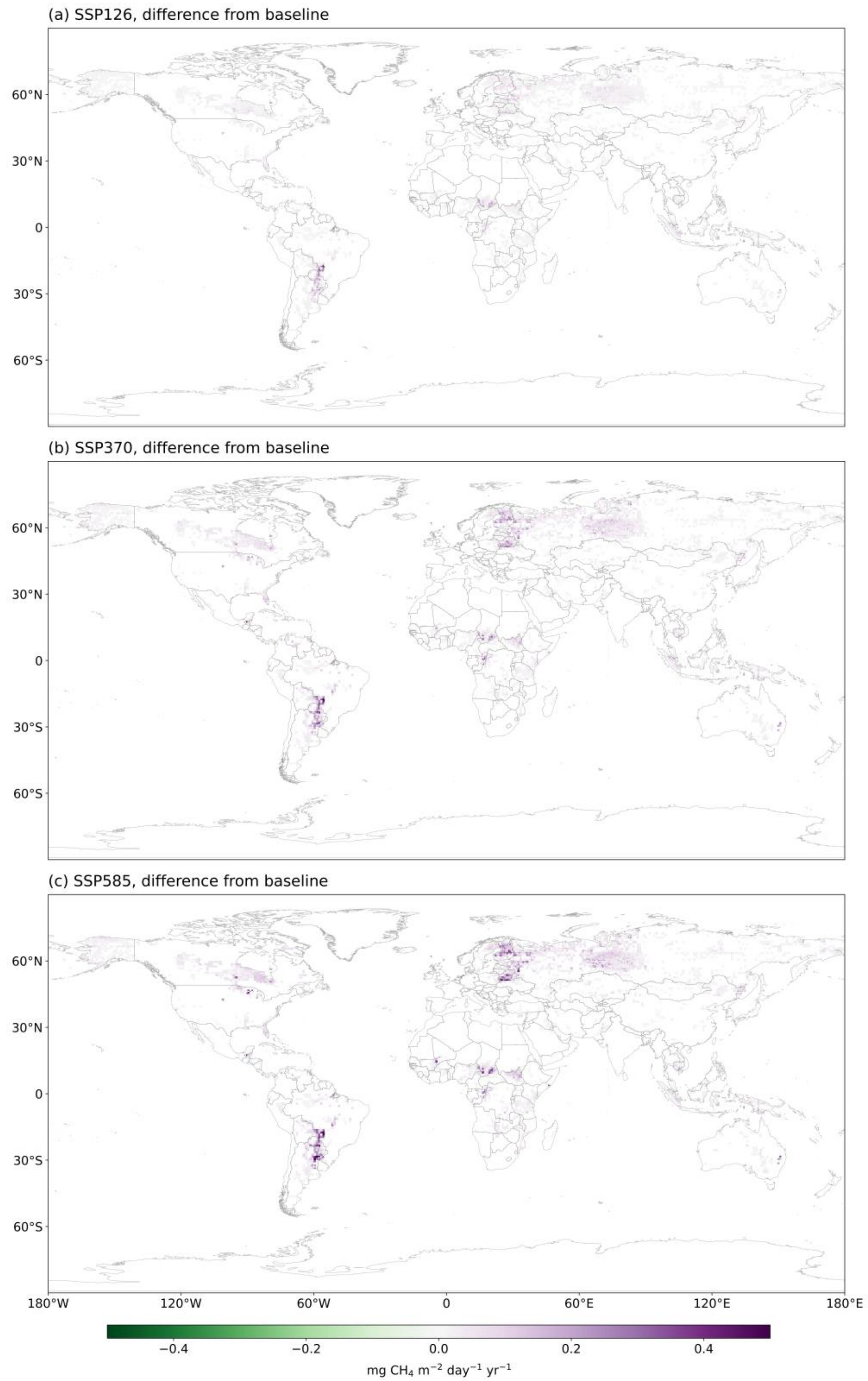

Figure S8 Uncertainty of GCMs in simulating Future trends of global wetland methane emissions from 1995 to 2100 under SSP126, SSP370, and SSP585.

Table S1. Site description in this study

No.	Site Name	Longitude	Latitude	Source	Reference
1	Cuini	-64.10	-0.81	Chamber	(Belger et al., 2011)
2	Itu	-63.56	-0.29	Chamber	(Belger et al., 2011)
3	EARTH	-83.57	10.22	Chamber	(Nahlik & Mitsch, 2011)
4	La Selva	-84.01	10.42	Chamber	(Nahlik & Mitsch, 2011)
5	Palo Verde	-85.33	10.34	Chamber	(Nahlik & Mitsch, 2011)
6	SSA-fen	-104.62	53.80	Chamber	(Sellers et al., 1997)
7	NSA-fen	-98.42	55.92	Chamber	(Sellers et al., 1997)
8	Sallie's Fen	-71.06	43.21	Chamber	(Zhuang & Crill, 2008)
9	Buck Hollow Bog	-84.02	42.45	Chamber	(Shannon & White, 1994)
10	Minnesota peatland1	-93.47	47.53	Chamber	(Clement et al., 1995; Shurpali et al., 1993; Shurpali & Verma, 1998)
11	Mer Bleue Bog	-75.48	45.41	Chamber	(Moore et al., 2011)
12	Minnesota peatland2	-93.47	47.53	Chamber	(Dise, 1993)
13	Stordalen1	19.05	68.33	Chamber	(Svensson et al., 1999)
14	Stordalen2	19.05	68.33	Chamber	(Jackowicz-Korczyński et al., 2010)
15	Degero Stormyr	19.55	64.18	Chamber	(Granberg et al., 2001)
16	Salmisue mire	30.93	62.78	Chamber	(Saarnio et al., 1997)
17	Ruovesi	24.02	81.83	Chamber	(Rinne et al., 2007)
18	Quebec1	-78.77	53.90	Chamber	(Pelletier et al., 2007)
19	Quebec2	-77.72	53.63	Chamber	(Pelletier et al., 2007)
20	Quebec3	-76.13	53.57	Chamber	(Pelletier et al., 2007)
21	Sanjiang Plain1	133.52	47.58	Chamber	(Huang et al., 2010; Song et al., 2009)
22	Sanjiang Plain2	133.52	47.58	Chamber	(Cui, 1997; Ding et al., 2004; Wang et al., 2002;

					YANG et al., 2006)
23	Loch Vale	-105.65	40.28	Chamber	(Wickland et al., 2001)
24	Ryans 1 Billagong	146.97	-36.12	Chamber	(Boon & Mitchell, 1995)
25	Tobolsk	68.12	58.47	Chamber	(Glagolev et al., 2011)
26	Surgut	73.33	61.43	Chamber	(Glagolev et al., 2011)
27	Pangody	74.96	65.87	Chamber	(Glagolev et al., 2011)
28	Plotnikovo	82.85	56.85	Chamber	(Glagolev et al., 2011)
29	Noyabrsk-Hills	74.49	63.12	Chamber	(Glagolev et al., 2011)
30	Noyabrsk-Palsa	75.55	63.80	Chamber	(Glagolev et al., 2011)
31	Vah	70.42	59.74	Chamber	(Glagolev et al., 2011)
32	Muhrino	68.70	60.89	Chamber	(Glagolev et al., 2011)
33	Tazovsky	78.92	67.18	Chamber	(Glagolev et al., 2011)
34	Gyda	78.55	70.89	Chamber	(Glagolev et al., 2011)
35	Skala	81.78	55.40	Chamber	(Glagolev et al., 2011)
36	BR-Npw	−56.41	−16.50	FluxnetCH_4	(Vourlitis et al., 2020)
37	BW-Gum	22.37	−18.96	FluxnetCH_4	(Helfter, 2020a)
38	BW-Nxr	23.18	−19.55	FluxnetCH_4	(Helfter, 2020b)
39	CA-SCB	-121.30	61.31	FluxnetCH_4	(Sonnentag & Helbig, 2020a)
40	CA-SCC	−121.30	61.31	FluxnetCH_4	(Sonnentag & Helbig, 2020b)
41	DE-Hte	12.18	54.21	FluxnetCH_4	(Koebsch & Jurasinski, 2020)
42	DE-Zrk	12.89	53.88	FluxnetCH_4	(Sachs et al., 2020)
43	DE-SfN	11.33	47.81	FluxnetCH_4	(Schmid & Klatt, 2020)
44	FI-Lom	24.21	68.00	FluxnetCH_4	(Lohila et al., 2020)
45	FI-Si2	24.20	61.84	FluxnetCH_4	(Vesala et al., 2020)
46	FI-Sii	24.19	61.83	FluxnetCH_4	(Vesala et al., 2020)
47	FR-LGt	2.28	47.32	FluxnetCH_4	(Jacotot et al., 2020)

48	ID-Pag	113.9	−2.32	FluxnetCH_4	(Sakabe et al., 2020)
49	JP-BBY	141.81	43.32	FluxnetCH_4	(Ueyama et al., 2020)
50	MY-MLM	111.15	1.45	FluxnetCH_4	(Wong et al., 2020)
51	NZ-Kop	175.55	−37.39	FluxnetCH_4	(Campbell & Goodrich, 2020)
52	RU-Ch2	161.35	68.62	FluxnetCH_4	(Goeckede, 2020)
53	RU-Cok	147.49	70.83	FluxnetCH_4	(Dolman et al., 2020)
54	SE-Deg	19.56	64.18	FluxnetCH_4	(Nilsson & Peichl, 2020)
55	US-A03	-149.88	70.50	FluxnetCH_4	(Billesbach & Sullivan, 2019a)
56	US-ICs	−149.31	68.61	FluxnetCH_4	(Euskirchen et al., 2020)
57	US-A10	-156.61	71.32	FluxnetCH_4	(Billesbach & Sullivan, 2019b)
58	US-Atq	−157.41	70.47	FluxnetCH_4	(Zona & Oechel, 2020a)
59	US-Beo	−156.61	71.28	FluxnetCH_4	(Zona & Oechel, 2020b)
60	US-Bes	−156.60	71.29	FluxnetCH_4	(Zona & Oechel, 2020c)
61	US-NGB	-156.61	71.28	FluxnetCH_4	(Torn & Dengel, 2018)
62	US-BZB	−148.32	64.70	FluxnetCH_4	(Euskirchen & Edgar, 2020a)
63	US-BZF	−148.31	64.70	FluxnetCH_4	(Euskirchen & Edgar, 2020b)
64	US-Uaf	-147.86	64.87	FluxnetCH_4	(Ueyama et al., 2018)
65	US-DPW	-81.44	28.05	FluxnetCH_4	(Hinkle, 2016)
66	US-Ivo	-155.75	68.49	FluxnetCH_4	(Zona & Oechel, 2016)
67	US-EDN	-122.11	37.62	FluxnetCH_4	(Oikawa, 2016)
68	US-EML	-149.25	63.88	FluxnetCH_4	(Schuur, 2018)
67	US-LA2	-90.29	29.86	FluxnetCH_4	(Krauss, 2019)
68	US-Los	-89.98	46.08	FluxnetCH_4	(Desai, 2016)
69	US-Myb	-121.76	38.05	FluxnetCH_4	(Matthes et al., 2016)
70	US-Sne	-121.75	38.04	FluxnetCH_4	(Shortt et al., 2020)
71	US-Tw1	-121.65	38.11	FluxnetCH_4	(Valach et al., 2016)
72	US-Tw4	−121.64	38.10	FluxnetCH_4	(Eichelmann et al., 2020)
73	US-Tw5	−121.64	38.11	FluxnetCH_4	(Valach et al., 2020)

74	US-NC4	−75.90	35.79	$FluxnetCH_4$	(Eichelmann et al., 2020)
75	US-NGC	−163.70	64.86	$FluxnetCH_4$	(Torn & Dengel, 2020)
76	US-ORv	-83.02	40.02	$FluxnetCH_4$	(Bohrer, 2016)
77	US-OWC	-82.51	41.38	$FluxnetCH_4$	(Bohrer & Kerns, 2018)
78	US-WPT	-83.00	41.46	$FluxnetCH_4$	(Chen & Chu, 2016)
79	US-EDN	-122.11	37.62	$FluxnetCH_4$	(Oikawa, 2016)
80	US-Stj	-75.44	39.09	$FluxnetCH_4$	(Vargas, 2018)
81	US-Srr	-122.03	38.2	$FluxnetCH_4$	(Bergamaschi & Windham-Myers, 2018)
82	US-LA1	-90.44	29.5	$FluxnetCH_4$	(Holm et al., 2020)

Table S2. The range of candidate and selected hyperparameters.

Model	Parameters	Search Range	Selected
DT	max_depth	[None, 10, 15, 30, 45, 60]	15
	min_samples_split	[2, 4, 6, 8, 10, 12]	12
RF	n_estimators	[50, 100, 200, 400, 600]	50
	max_depth	[None, 10, 15, 30, 45, 60]	10
	min_samples_split	[4, 6, 8, 10, 12]	6
XGB	n_estimators	[50, 100, 200, 250]	50
	max_depth	[None, 10, 15, 30, 45, 60]	None
	learning_rate	[0.05, 0.1, 0.2, 0.3]	0.05
ANN	neurons	[16, 32, 64, 128, 256, 512]	[256, 128, 64, 32]
	layers	[2, 3, 4, 5]	4
	epochs	[1000, 1500, 2000, 3000]	1000
	activation function	[‘ReLU’, ‘tanh’]	ReLU
GRU	neurons	[16, 32, 64, 128, 256, 512]	[256, 128, 64]
	layers	[2, 3, 4, 5]	3
	epochs	[1000, 1500, 2000, 3000]	2000
	activation function	[‘ReLU’, ‘tanh’]	ReLU
LSTM	neurons	[16, 32, 64, 128, 256, 512]	[256, 128]
	layers	[2, 3, 4, 5]	2
	epochs	[1000, 1500, 2000, 3000]	2000
	activation function	[‘ReLU’, ‘tanh’]	ReLU

Table S3. The percentage that future conditions will exceed the variable space of training data

		PREC	TAIR	SOLAR	rlds	RH
ERA5	1979-2022	0%	0%	0%	0%	0%
gfdl-esm4	historical	0%	0.0%	0%	0.3%	0%
	SSP126	0%	0.0%	0%	2.1%	0%
	SSP370	0%	0.1%	0%	5.7%	0%
	SSP585	0%	0.2%	0%	6.5%	0%
ipsl-cm6a-lr	historical	0%	0.0%	0%	0.3%	0%
	SSP126	0%	0.0%	0%	3.2%	0%
	SSP370	0%	0.4%	0%	8.2%	0%
	SSP585	0%	1.7%	0%	10.3%	0%
mpi-esm1-2-hr	historical	0%	0.0%	0%	0.2%	0%
	SSP126	0%	0.0%	0%	1.5%	0%
	SSP370	0%	0.0%	0%	4.9%	0%
	SSP585	0%	0.1%	0%	5.8%	0%
mri-esm2-0	historical	0%	0.0%	0%	0.2%	0%
	SSP126	0%	0.0%	0%	2.5%	0%
	SSP370	0%	0.1%	0%	6.0%	0%
	SSP585	0%	0.4%	0%	7.6%	0%
ukesm1-0-ll	historical	0%	0.0%	0%	0.4%	0%
	SSP126	0%	0.0%	0%	5.3%	0%
	SSP370	0%	1.4%	0%	10.5%	0%
	SSP585	0%	3.5%	0%	12.3%	0%

Table S4. The range of climate variables

		TAIR	PREC	SOLAR	rlds	RH
In situ		[-35.34, 33.61]	[0.0, 1093.96]	[-6.09, 450.82]	[146.98, 432.45]	[17.35, 100.25]
ERA5	1979-2022	[-20.72, 32.31]	[0.19, 1803.53]	[64.77, 300.04]	[149.01, 435.51]	[19.09, 100.89]
gfdl-esm4	historical	[-21.5, 31.26]	[0.15, 770.81]	[68.55, 298.21]	[180.36, 443.75]	[22.97, 92.21]
	SSP126	[-20.55, 32.42]	[0.05, 866.74]	[64.36, 303.78]	[185.33, 452.66]	[21.13, 92.28]
	SSP370	[-19.9, 35.58]	[0.1, 899.34]	[61.53, 303.46]	[184.59, 471.82]	[21.03, 91.83]
	SSP585	[-20.96, 35.79]	[0.08, 895.87]	[61.5, 300.09]	[184.09, 477.55]	[21.45, 91.89]
ipsl-cm6a-lr	historical	[-21.82, 31.84]	[0.2, 1000.46]	[68.88, 299.07]	[182.12, 445.45]	[22.11, 92.63]
	SSP126	[-21.22, 32.93]	[0.04, 938.37]	[65.72, 297.44]	[183, 449.39]	[20.69, 93.14]
	SSP370	[-20.62, 37.17]	[0.23, 1038.4]	[60.66, 297.6]	[186.42, 479.7]	[19.75, 93.22]
	SSP585	[-20.4, 39.18]	[0.09, 1114.95]	[58.75, 298.04]	[186.37, 496.83]	[19.52, 92.82]
mpi-esm1-2-hr	historical	[-22.54, 31.45]	[0.3, 1002.48]	[67.59, 299.52]	[178.39, 443.99]	[23.07, 93.8]
	SSP126	[-20.16, 32.51]	[0.04, 995.93]	[68.63, 301.01]	[186.37, 448.52]	[23.4, 93.59]
	SSP370	[-20.41, 35]	[0.06, 952.14]	[63.79, 300.35]	[185.77, 467.43]	[22.94, 93.57]
	SSP585	[-20.64, 36.6]	[0.03, 957.38]	[63.74, 298.04]	[187.21, 474.92]	[22.06, 93.2]
mri-esm2-0	historical	[-21.54, 31.38]	[0.08, 845.73]	[70.58, 299.48]	[181.14, 442.77]	[23.57, 92.36]
	SSP126	[-20.55, 33.11]	[0.1, 863.76]	[66.77, 304.69]	[186.09, 452.08]	[21.6, 91.83]
	SSP370	[-20.37, 35.52]	[0.05, 862.94]	[64.13, 298.45]	[188.2, 470.25]	[19.72, 91.39]
	SSP585	[-19.65, 37.24]	[0.07, 960.96]	[63.13, 302.11]	[190.94, 476.39]	[19.63, 91.84]
ukesm1-0-ll	historical	[-21.54, 31.66]	[0.1, 783.45]	[65, 296.14]	[178.07, 444.91]	[21.65, 91.9]
	SSP126	[-20.15, 34.07]	[0.11, 816.8]	[65.24, 309.14]	[189.96, 459.28]	[21.57, 92.33]
	SSP370	[-19.83, 38.05]	[0.04, 985.31]	[63.04, 303.39]	[186.72, 488.42]	[21.05, 92.35]
	SSP585	[-19.56, 39.97]	[0.1, 958.82]	[59.2, 310.59]	[186.96, 499.08]	[18.45, 92.29]

Reference:

Belger, L., Forsberg, B. R., & Melack, J. M. (2011). Carbon dioxide and methane emissions from interfluvial wetlands in the upper Negro River basin, Brazil. *Biogeochemistry*, *105*(1), 171–183. https://doi.org/10.1007/s10533-010-9536-0

Bergamaschi, B., & Windham-Myers, L. (2018). AmeriFlux US-Srr Suisun marsh-Rush Ranch [Data set]. https://doi.org/10.17190/AMF/1418685

Billesbach, D., & Sullivan, R. (2019a). AmeriFlux US-A03 ARM-AMF3-Oliktok [Data set]. https://doi.org/10.17190/AMF/1498752

Billesbach, D., & Sullivan, R. (2019b). AmeriFlux US-A10 ARM-NSA-Barrow [Data set]. https://doi.org/10.17190/AMF/1498753

Bohrer, G. (2016). AmeriFlux US-ORv Olentangy River Wetland Research Park [Data set]. https://doi.org/10.17190/AMF/1246135

Bohrer, G., & Kerns, J. (2018). AmeriFlux US-OWC Old Woman Creek [Data set]. https://doi.org/10.17190/AMF/1418679

Boon, P. I., & Mitchell, A. (1995). Methanogenesis in the sediments of an Australian freshwater wetland: Comparison with aerobic decay, and factors controlling methanogenesis. *FEMS Microbiology Ecology*, *18*(3), 175–190. https://doi.org/10.1111/j.1574-6941.1995.tb00175.x

Campbell, D., & Goodrich, J. (2020). FLUXNET-CH4 NZ-Kop Kopuatai, New Zealand [Data set]. FLUXNET-CH4 Community Product. https://doi.org/10.18140/FLX/1669652

Chen, J., & Chu, H. (2016). AmeriFlux US-WPT Winous Point North Marsh [Data set]. https://doi.org/10.17190/AMF/1246155

Clement, R. J., Verma, S. B., & Verry, E. S. (1995). Relating chamber measurements to eddy correlation measurements of methane flux. *Journal of Geophysical Research: Atmospheres*, *100*(D10), 21047–21056. https://doi.org/10.1029/95JD02196

Cui, B. (1997). Estimation of CH4 emission from Sanjiang plain. *Scientia Geographica Sinica*, *17*, 93–95.

Desai, A. (2016). AmeriFlux US-Los Lost Creek [Data set]. https://doi.org/10.17190/AMF/1246071

Ding, W., Cai, Z., & Wang, D. (2004). Preliminary budget of methane emissions from natural wetlands in China. *Atmospheric Environment*, *38*(5), 751–759. https://doi.org/10.1016/j.atmosenv.2003.10.016

Dise, Nancy. B. (1993). Methane emission from Minnesota peatlands: Spatial and seasonal variability. *Global Biogeochemical Cycles*, *7*(1), 123–142. https://doi.org/10.1029/92GB02299

Dolman, H., Maximox, T., Parmentier, F. J., Budishev, A., & Marchesini, L. B. (2020). FLUXNET-CH4 RU-Cok Chokurdakh, Russian Federation [Data set]. FLUXNET-CH4 Community Product. https://doi.org/10.18140/FLX/1669656

Eichelmann, E., Knox, S., Sanchez, C. R., Valach, A., Sturtevant, C., Szutu, D., et al. (2020). FLUXNET-CH4 US-Tw4 Twitchell East End Wetland, United States [Data set]. FLUXNET-CH4 Community Product. https://doi.org/10.18140/FLX/1669698

Euskirchen, E., & Edgar, C. (2020a). FLUXNET-CH4 US-BZB Bonanza Creek Thermokarst Bog, United States [Data set]. FLUXNET-CH4 Community Product. https://doi.org/10.18140/FLX/1669668

Euskirchen, E., & Edgar, C. (2020b). FLUXNET-CH4 US-BZF Bonanza Creek Rich Fen, United States [Data set]. FLUXNET-CH4 Community Product. https://doi.org/10.18140/FLX/1669669

Euskirchen, E., Bret-Harte, M., & Edgar, C. (2020). FLUXNET-CH4 US-ICs Imnavait Creek Watershed Wet Sedge Tundra, United States [Data set]. FLUXNET-CH4 Community Product. https://doi.org/10.18140/FLX/1669678

Glagolev, M., Kleptsova, I., Filippov, I., Maksyutov, S., & Machida, T. (2011). Regional methane emission from West Siberia mire landscapes. *Environmental Research Letters*, *6*(4), 045214. https://doi.org/10.1088/1748-9326/6/4/045214

Goeckede, M. (2020). FLUXNET-CH4 RU-Ch2 Chersky reference, Russian Federation [Data set]. FLUXNET-CH4 Community Product. https://doi.org/10.18140/FLX/1669654

Granberg, G., Ottosson-Löfvenius, M., Grip, H., Sundh, I., & Nilsson, M. (2001). Effect of climatic variability from 1980 to 1997 on simulated methane emission from a boreal mixed mire in northern Sweden. *Global Biogeochemical Cycles*, *15*(4), 977–991. https://doi.org/10.1029/2000GB001356

Helfter, C. (2020a). FLUXNET-CH4 BW-Gum Guma, Botswana [Data set]. FLUXNET-CH4 Community Product. https://doi.org/10.18140/FLX/1669370

Helfter, C. (2020b). FLUXNET-CH4 BW-Nxr Nxaraga, Botswana [Data set]. FLUXNET-CH4 Community Product. https://doi.org/10.18140/FLX/1669518

Hinkle, C. R. (2016). AmeriFlux US-DPW Disney Wilderness Preserve Wetland [Data set]. https://doi.org/10.17190/AMF/1562387

Holm, G. O., Perez, B. C., McWhorter, D. E., Krauss, K. W., Raynie, R. C., & Killebrew, C. J. (2020). FLUXNET-CH4 US-LA1 Pointe-aux-Chenes Brackish Marsh, United States [Data set]. FLUXNET-CH4 Community Product. https://doi.org/10.18140/FLX/1669680

Huang, Y., Sun, W., Zhang, W., Yu, Y., Su, Y., & Song, C. (2010). Marshland conversion to cropland in northeast China from 1950 to 2000 reduced the greenhouse effect. *Global Change Biology*, *16*(2), 680–695.

Jackowicz-Korczyński, M., Christensen, T. R., Bäckstrand, K., Crill, P., Friborg, T., Mastepanov, M., & Ström, L. (2010). Annual cycle of methane emission from a subarctic peatland. *Journal of Geophysical Research: Biogeosciences*, *115*(G2). https://doi.org/10.1029/2008JG000913

Jacotot, A., Gogo, S., & Laggoun-Défarge, F. (2020). FLUXNET-CH4 FR-LGt La Guette, France [Data set]. FLUXNET-CH4 Community Product. https://doi.org/10.18140/FLX/1669641

Koebsch, F., & Jurasinski, G. (2020). FLUXNET-CH4 DE-Hte Huetelmoor, Germany [Data set]. FLUXNET-CH4 Community Product. https://doi.org/10.18140/FLX/1669634

Krauss, K. (2019). AmeriFlux US-LA2 Salvador WMA Freshwater Marsh [Data set]. https://doi.org/10.17190/AMF/1543387

Lohila, A., Aurela, M., Tuovinen, J.-P., Laurila, T., Hatakka, J., Rainne, J., & Mäkelä, T. (2020). FLUXNET-CH4 FI-Lom Lompolojankka, Finland [Data set]. FLUXNET-CH4 Community Product. https://doi.org/10.18140/FLX/1669638

Matthes, J. H., Sturtevant, C., Oikawa, P., Chamberlain, S. D., Szutu, D., Arias-Ortiz, A., et al. (2016). AmeriFlux US-Myb Mayberry Wetland [Data set]. https://doi.org/10.17190/AMF/1246139

Moore, T. R., De Young, A., Bubier, J. L., Humphreys, E. R., Lafleur, P. M., & Roulet, N. T. (2011). A Multi-Year Record of Methane Flux at the Mer Bleue Bog, Southern Canada. *Ecosystems*, *14*(4), 646–657. https://doi.org/10.1007/s10021-011-9435-9

Nahlik, A. M., & Mitsch, W. J. (2011). Methane emissions from tropical freshwater wetlands located in different climatic zones of Costa Rica. *Global Change Biology*, *17*(3), 1321–1334. https://doi.org/10.1111/j.1365-2486.2010.02190.x

Nilsson, M. B., & Peichl, M. (2020). FLUXNET-CH4 SE-Deg Degero, Sweden [Data set]. FLUXNET-CH4 Community Product. https://doi.org/10.18140/FLX/1669659

Oikawa, P. (2016). AmeriFlux US-EDN Eden Landing Ecological Reserve [Data set]. https://doi.org/10.17190/AMF/1543381

Pelletier, L., Moore, T. R., Roulet, N. T., Garneau, M., & Beaulieu-Audy, V. (2007). Methane fluxes from three peatlands in the La Grande Rivière watershed, James Bay lowland, Canada. *Journal of Geophysical Research: Biogeosciences*, *112*(G1). https://doi.org/10.1029/2006JG000216

Rinne, J., Riutta, T., Pihlatie, M., Aurela, M., Haapanala, S., Tuovinen, J.-P., et al. (2007). Annual cycle of methane emission from a boreal fen measured by the eddy covariance technique. *Tellus B: Chemical and Physical Meteorology*, *59*(3), 449–457. https://doi.org/10.1111/j.1600-0889.2007.00261.x

Saarnio, S., Alm, J., Silvola, J., Lohila, A., Nykänen, H., & Martikainen, P. J. (1997). Seasonal variation in CH4 emissions and production and oxidation potentials at microsites on an oligotrophic pine fen. *Oecologia*, *110*(3), 414–422. https://doi.org/10.1007/s004420050176

Sachs, T., Wille, C., Larmanou, E., & Franz, D. (2020). FLUXNET-CH4 DE-Zrk Zarnekow, Germany [Data set]. FLUXNET-CH4 Community Product. https://doi.org/10.18140/FLX/1669636

Sakabe, A., Itoh, M., Hirano, T., & Kusin, K. (2020). FLUXNET-CH4 ID-Pag Palangkaraya undrained forest, Indonesia [Data set]. FLUXNET-CH4 Community Product. https://doi.org/10.18140/FLX/1669643

Schmid, H. P., & Klatt, J. (2020). FLUXNET-CH4 DE-SfN Schechenfilz Nord, Germany [Data set]. FLUXNET-CH4 Community Product. https://doi.org/10.18140/FLX/1669635

Schuur, T. (2018). AmeriFlux US-EML Eight Mile Lake Permafrost thaw gradient, Healy Alaska. [Data set]. https://doi.org/10.17190/AMF/1418678

Sellers, P. J., Hall, F. G., Kelly, R. D., Black, A., Baldocchi, D., Berry, J., et al. (1997). BOREAS in 1997: Experiment overview, scientific results, and future directions. *Journal of Geophysical Research: Atmospheres*, *102*(D24), 28731–28769. https://doi.org/10.1029/97JD03300

Shannon, R. D., & White, J. R. (1994). A Three-Year Study of Controls on Methane Emissions from Two Michigan Peatlands. *Biogeochemistry*, *27*(1), 35–60.

Shortt, R., Hemes, K., Szutu, D., Verfaillie, J., & Baldocchi, D. (2020). FLUXNET-CH4 US-Sne Sherman Island Restored Wetland, United States [Data set]. FLUXNET-CH4 Community Product. https://doi.org/10.18140/FLX/1669693

Shurpali, N. J., & Verma, S. B. (1998). Micrometeorological measurements of methane flux in a Minnesota peatland during two growing seasons. *Biogeochemistry*, *40*(1), 1–15. https://doi.org/10.1023/A:1005875307146

Shurpali, N. J., Verma, S. B., Clement, R. J., & Billesbach, D. P. (1993). Seasonal distribution of methane flux in a Minnesota peatland measured by eddy correlation. *Journal of Geophysical Research: Atmospheres*, *98*(D11), 20649–20655. https://doi.org/10.1029/93JD02181

Song, C., Xu, X., Tian, H., & Wang, Y. (2009). Ecosystem–atmosphere exchange of CH4 and N2O and ecosystem respiration in wetlands in the Sanjiang Plain, Northeastern China. *Global Change Biology*, *15*(3), 692–705. https://doi.org/10.1111/j.1365-2486.2008.01821.x

Sonnentag, O., & Helbig, M. (2020a). FLUXNET-CH4 CA-SCB Scotty Creek Bog, Canada [Data set]. FLUXNET-CH4 Community Product. https://doi.org/10.18140/FLX/1669613

Sonnentag, O., & Helbig, M. (2020b). FLUXNET-CH4 CA-SCC Scotty Creek Landscape, Canada [Data set]. FLUXNET-CH4 Community Product. https://doi.org/10.18140/FLX/1669628

Svensson, B. H., Christensen, T. R., Johansson, E., & Öquist, M. (1999). Interdecadal Changes in CO2 and CH4 Fluxes of a Subarctic Mire: Stordalen Revisited after 20 Years. *Oikos*, *85*(1), 22–30. https://doi.org/10.2307/3546788

Torn, M., & Dengel, S. (2018). AmeriFlux US-NGB NGEE Barrow [Data set]. https://doi.org/10.17190/AMF/1436326

Torn, M., & Dengel, S. (2020). FLUXNET-CH4 US-NGC NGEE Arctic Council, United States [Data set]. FLUXNET-CH4 Community Product. https://doi.org/10.18140/FLX/1669688

Ueyama, M., Iwata, H., & Harazono, Y. (2018). AmeriFlux US-Uaf University of Alaska, Fairbanks [Data set]. https://doi.org/10.17190/AMF/1480322

Ueyama, M., Hirano, T., & Kominami, Y. (2020). FLUXNET-CH4 JP-BBY Bibai bog, Japan [Data set]. FLUXNET-CH4 Community Product. https://doi.org/10.18140/FLX/1669646

Valach, A., Shortt, R., Szutu, D., Eichelmann, E., Knox, S., Hemes, K., et al. (2016). AmeriFlux US-Tw1 Twitchell Wetland West Pond [Data set]. https://doi.org/10.17190/AMF/1246147

Valach, A., Kasak, K., Szutu, D., Verfaillie, J., & Baldocchi, D. (2020). FLUXNET-CH4 US-Tw5 East Pond Wetland, United States [Data set]. FLUXNET-CH4 Community Product. https://doi.org/10.18140/FLX/1669699

Vargas, R. (2018). AmeriFlux US-StJ St Jones Reserve [Data set]. https://doi.org/10.17190/AMF/1480316

Vesala, T., Tuittila, E.-S., Mammarella, I., & Alekseychik, P. (2020). FLUXNET-CH4 FI-Si2 Siikaneva-2 Bog, Finland [Data set]. FLUXNET-CH4 Community Product. https://doi.org/10.18140/FLX/1669639

Vourlitis, G., Dalmagro, H., de S Nogueira, J., Johnson, M., & Arruda, P. (2020). FLUXNET-CH4 BR-Npw Northern Pantanal Wetland, Brazil [Data set]. FLUXNET-CH4 Community Product. https://doi.org/10.18140/FLX/1669368

Wang, D., Lu, X., Ding, W., Cai, Z., & Wang, Y. (2002). Comparison of methane emission from marsh and paddy field in Sanjiang Plain. *Scientia Geographica Sinica*, *22*(4), 500–503.

Wickland, K. P., Striegl, R. G., Mast, M. A., & Clow, D. W. (2001). Carbon gas exchange at a southern Rocky Mountain wetland, 1996–1998. *Global Biogeochemical Cycles*, *15*(2), 321–335. https://doi.org/10.1029/2000GB001325

Wong, G. X., Melling, L., Tang, A. C. I., Aeries, E. B., Waili, J. W., Musin, K. K., et al. (2020). FLUXNET-CH4 MY-MLM Maludam National Park, Malaysia [Data set]. FLUXNET-CH4 Community Product. https://doi.org/10.18140/FLX/1669650

YANG, J.-S., LIU, J.-S., WANG, J.-D., YU, J.-B., SUN, Z.-G., & LI, X.-H. (2006). Emissions of CH4 and N2O from a wetland in the Sanjiang Plain. *Chinese Journal of Plant Ecology*, *30*(3), 432.

Zhuang, Q., & Crill, P. (2008). NCEAS 10645: Toward an adequate quantification of CH4 emissions from land ecosystems: Integrating field and in-situ observations, satellite data, and modeling. Sallies Fen NH CH4 Flux 1994–2001. *National Center for Ecological Analysis and Synthesis and Stockholm University*.

Zona, D., & Oechel, W. (2016). AmeriFlux US-Ivo Ivotuk [Data set]. https://doi.org/10.17190/AMF/1246067

Zona, D., & Oechel, W. C. (2020a). FLUXNET-CH4 US-Atq Atqasuk, United States [Data set]. FLUXNET-CH4 Community Product. https://doi.org/10.18140/FLX/1669663

Zona, D., & Oechel, W. C. (2020b). FLUXNET-CH4 US-Beo Barrow Environmental Observatory (BEO) tower, United States [Data set]. FLUXNET-CH4 Community Product. https://doi.org/10.18140/FLX/1669664

Zona, D., & Oechel, W. C. (2020c). FLUXNET-CH4 US-Bes Barrow-Bes (Biocomplexity Experiment South tower), United States [Data set]. FLUXNET-CH4 Community Product. https://doi.org/10.18140/FLX/1669665
